# Multiomic profiling of canine immune and metabolic aging in a cohort of young and senior beagles

**DOI:** 10.3389/fimmu.2026.1906638

**Published:** 2026-09-08

**Authors:** Abdullah Abood, Artemis S. Louyakis, Matthew Jackson, Rachel Eichman, Regina Hollar, Kiran S. Panickar, Jeffrey Brockman

**Affiliations:** Science and Technology Center, Hill’s Pet Nutrition, Inc., Topeka, KS, United States

**Keywords:** bulk RNAseq, canine aging, GWAS, single-cell RNAseq, untargeted metabolomics

## Abstract

**Introduction:**

Developing a comprehensive understanding of canine aging requires integration of immune, metabolic, and genetic changes across molecular layers. While high-throughput omics have been widely applied to human aging, canine studies have typically used single modalities or small cohorts, limiting systems-level insight, particularly for immune and metabolic aging. To address this gap, we generated a multiomic immune and metabolic aging reference in a cohort of senior and young beagle dogs.

**Methods:**

We performed untargeted serum metabolomics in 50 canines (25 senior and 25 young). Additionally, we collected clinical biomarkers and measured gene expression from whole blood using bulk RNA sequencing (bulk RNAseq) from a subset of 39 canines (19 senior, 20 young). We also leveraged single-cell RNA sequencing (scRNAseq) from peripheral blood mononuclear cells (PBMCs) on a subset of 30 canines (15 senior, 15 young).

**Results:**

Obtaining ~318,000 PBMC transcriptomes—comprising the largest canine single-cell dataset to date—we resolved 27 distinct immune populations. Age-associated shifts were characterized by loss of naïve lymphocytes with expansion of selected myeloid and Th1-like effector subsets. This was reinforced by remodeling across the transcriptome and metabolome, including coordinated suppression of cell-cycle, DNA repair, and mitochondrial pathways in adaptive immune cells, enrichment of inflammatory signaling in innate immune cells, and depletion of ether-linked lipids and other membrane-associated lipids in serum. To demonstrate the utility of this reference, we leveraged external resources to prioritize a set of candidate genes, including *PLA2G4A* and *IRF4*, with potential roles in canine immune and metabolic aging.

**Discussion:**

These findings characterize the immunometabolic landscape of aging in canine blood, creating a blueprint for identifying interventions to promote healthy aging and longevity.

## Introduction

Biological aging is defined as a progressive decline in physiological integrity and homeostatic capacity, driven by the gradual accumulation of molecular damage and a subsequent susceptibility to disease (1). Lopez-Otin et al. (1) formalized this process into the ‘Hallmarks of Aging’ encompassing genomic instability, telomere attrition, epigenetic alterations, loss of proteostasis, deregulated nutrient-sensing, mitochondrial dysfunction, cellular senescence, stem cell exhaustion, and altered intercellular communication (1, 2). These interconnected mechanisms are broadly conserved across species and provide a useful framework for studying aging in canines (3, 4).

Among these hallmarks, altered intercellular communication, often manifesting as chronic, low-grade inflammation, or ‘inflammaging’, has emerged as a critical driver of age-related pathology (5). Immune aging is tightly coupled to metabolic reprogramming within immune cells, a crosstalk known as immunometabolism (6, 7). The immune system thereby acts both as a sensor of organismal stress and as a mediator of systemic decline, integrating signals from multiple tissues and contributing to the progression of multimorbidity (8, 9). In humans, system-level high-throughput omics approaches have been widely applied to aging, yielding large-scale profiles of age-associated changes in immune composition, gene expression, epigenetic state, and circulating metabolites (10, 11).

In contrast, canine aging has largely been studied using single modalities or modest cohort sizes, limiting the ability to build an integrated view of immune and metabolic remodeling (12–14). Prior work has begun to delineate the impact of aging on the canine immune system, reporting declines in adaptive immune populations (including CD4^+^ T cells and B cells) and increases in myeloid-derived suppressor like cells (MDSC like), along with the accumulation of immature neutrophils and senescent-like CD8^+^ T cells with mitochondrial dysfunction, leading to a state of immunosenescence (12, 15). In this state the immature neutrophils exhibit heightened intercellular communication. In addition, age-associated remodeling of developmental and metabolic gene programs has been observed, alongside changes in DNA methylation patterns and chromatin accessibility (13, 16).

**Figure 1:**
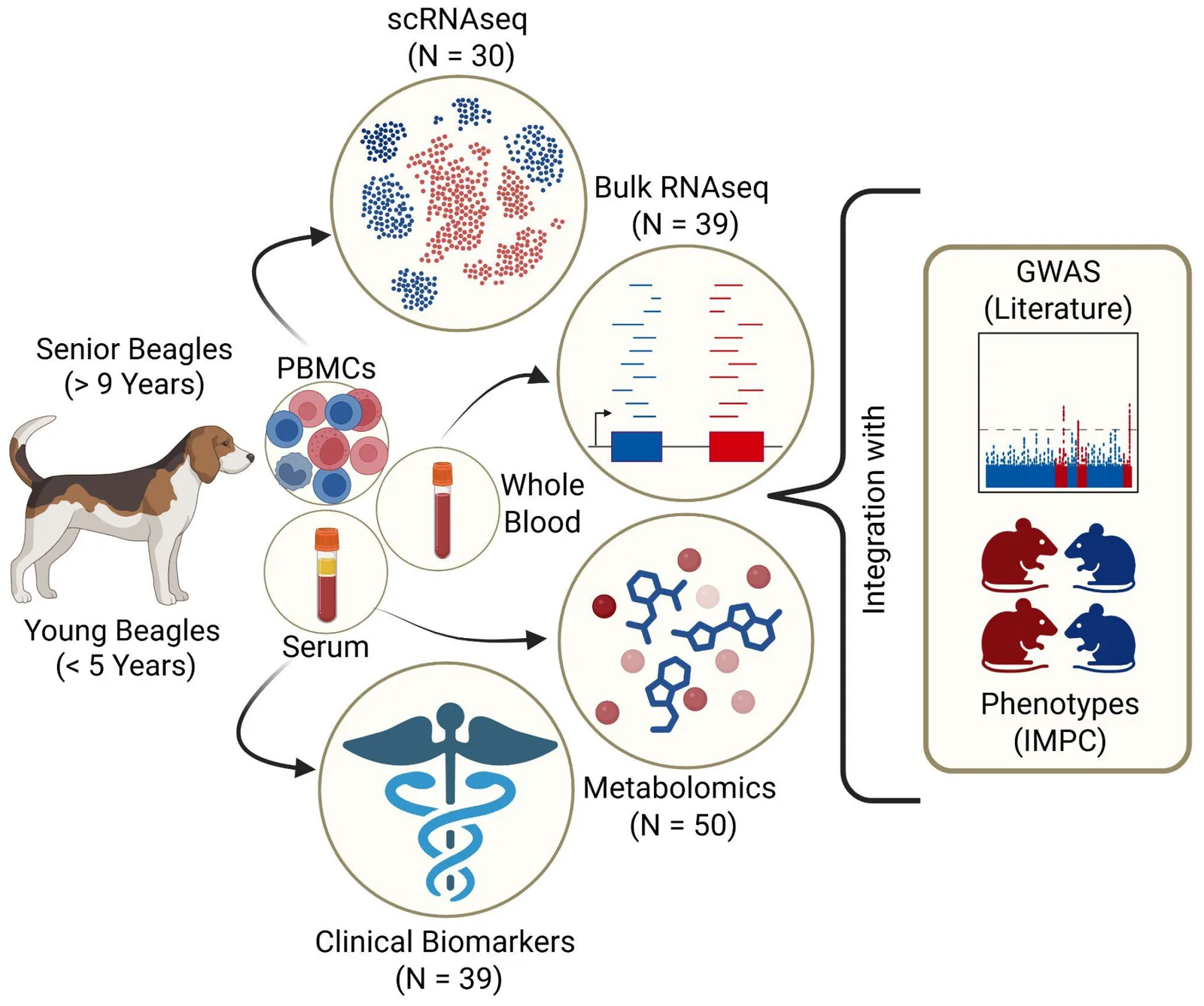
Schematic overview of the multi-omic study design and analytical workflow. PBMCs and serum were collected from a cohort of young (< 5 years) and senior (> 9 years) beagles. Samples underwent parallel processing for single-cell RNA sequencing (scRNA-seq; N = 30), bulk RNA sequencing (N = 39), serum metabolomics (N = 50), and clinical biomarker measurements (N = 39). These multimodal datasets were integrated with external canine and human GWAS data and murine knockout phenotype data (IMPC) to characterize age-associated molecular landscapes. PBMCs, peripheral blood mononuclear cells; IMPC, International Mouse Phenotyping Consortium, GWAS, genome-wide association studies.

To increase knowledge and understanding in the growing field of canine immunometabolism, we conducted a cross-scale multiomic study in a well-characterized cohort of healthy senior and young beagles (**Figure 1**). We measured serum metabolite abundance changes using untargeted metabolomics in 50 canines (25 senior and 25 young). In a subset of 39 canines (19 senior, 20 young), we performed complete blood counts (CBC) and serum chemistry screens along with whole blood gene expression profiling via bulk RNAseq. Additionally, we leveraged scRNAseq on a reduced subset of 30 canines (15 young and 15 senior). The latter yielded ~318,000 high quality single-cell transcriptomes, which, to our knowledge, represents the largest scRNAseq dataset to date (**Supplementary Table 1**) (64–70), and resolved 27 distinct immune populations. Furthermore, bulk transcriptomics and untargeted metabolomics identified broad age-associated remodeling, including 3,265 significant differentially expressed genes and 216 metabolites with significant differential abundance associated with aging related pathways.

Our results reinforce and extend the characteristic signatures of inflammaging and immunosenescence previously described in canines and other mammals. Leveraging these evolutionarily shared aging and longevity pathways across mammals (17) demonstrates the utility of integrating this reference with diverse external resources. Specifically, the findings were bridged with functional data from the International Mouse Phenotyping Consortium (IMPC) (18), evolutionary constraints from Zoonomia (19), and systematically implicated candidate genes from human and canine aging Genome Wide Association Studies (GWAS) (20–23).

Ultimately, this work establishes a multimodal reference of immune and metabolic aging in canine peripheral blood (isolated PBMCs and whole blood) and serum, providing a foundational resource to help guide the development of interventions aimed at improving health outcomes and extending the lifespan of canines.

## Results

### Aging skews canine PBMC composition toward myeloid expansion and loss of naive lymphocytes

**Figure 2:**
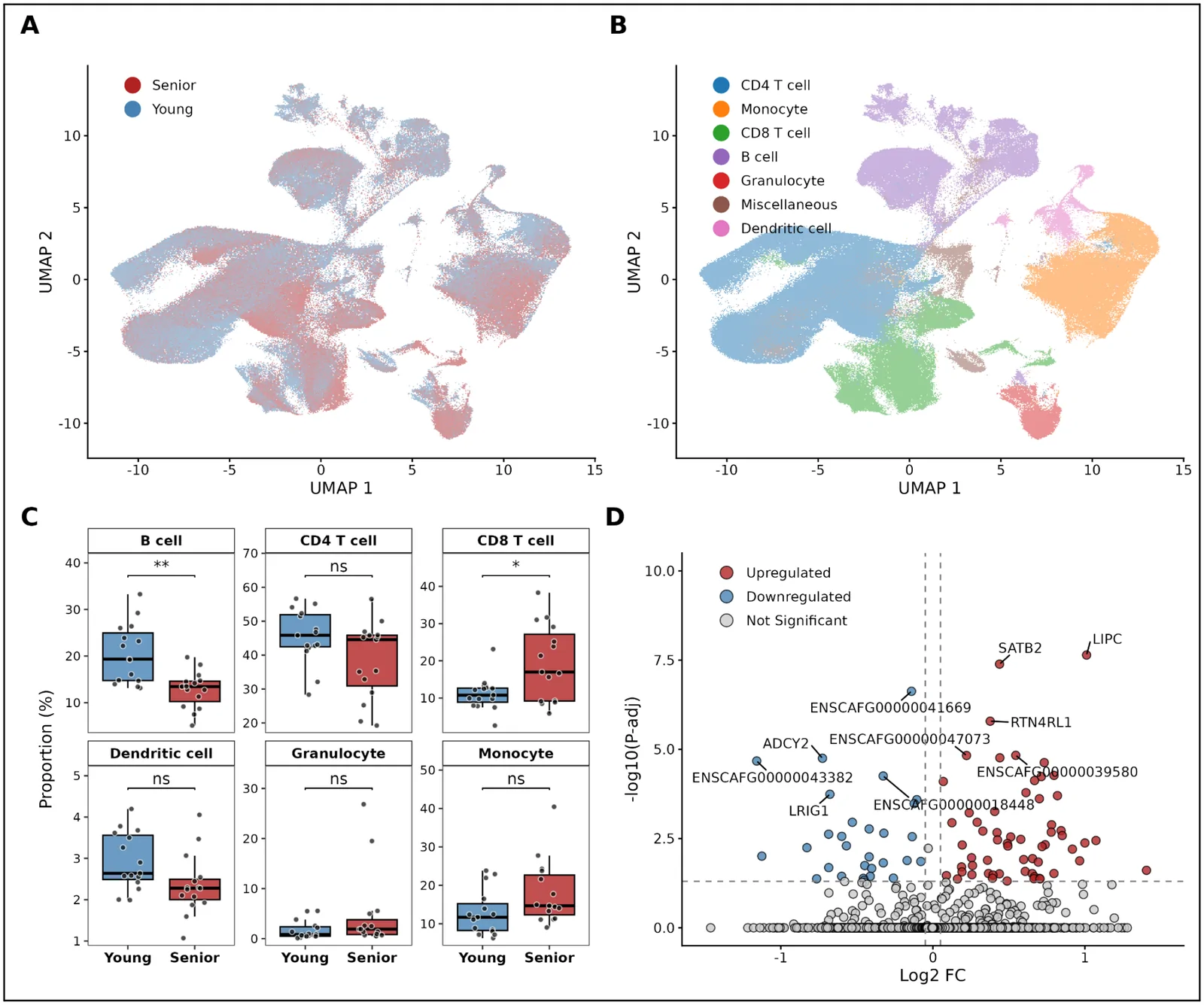
Single-cell transcriptional landscape and age-associated alterations in canine PBMCs. **(A)** Uniform Manifold Approximation and Projection (UMAP) embedding of ~318,000 cells integrated from 30 animals, colored by age group. **(B)** UMAP projection colored by major annotated cell lineages derived from Ammons et al. **(C)** Box plots quantifying changes in cell type proportions (as a percentage of total PBMCs) between Young (blue) and Senior (red) cohorts. Statistical significance was determined using propeller (speckle) with Benjamini–Hochberg FDR adjustment across cell types (ns, not significant, *FDR < 0.05, **FDR < 0.01). Young n = 15, Senior n = 15). **(D)** Volcano plot displaying pseudobulk differential expression results. Each dot represents a gene; red points denote genes significantly upregulated in Senior dogs, while blue points denote downregulated genes (adjusted *P*-value < 0.05, |log_2_FC| > 0.05). Top differentially expressed genes are labeled by gene symbol or Ensembl ID.

After quality control, we obtained ~318,000 high-quality PBMC transcriptomes with a median of 1,669 genes detected per cell (**Figures 2A–C**). Clustering resolved 27 transcriptionally distinct populations that we grouped into seven major immune lineages adhering to Ammons et al. (15): monocytes, granulocytes, CD4^+^ T cells, CD8^+^ T cells, B cells, dendritic cells, and miscellaneous including CD34+ unclassified, cycling T cell, DN T cell, gd T cell, and NK T cell (**Figures 2B, C**; **Supplementary Figure 1**). All compositional comparisons were performed at the individual level using per-canine lineage proportions (See Methods).

At the major-lineage level, senior canines showed an increased proportion of inflammatory innate immune cells, including nominal higher monocyte frequencies (p = 0.033, FDR = 0.076; ~18% in seniors vs ~13% in young), and a nominal trend toward more granulocytes (p = 0.070, FDR = 0.080; ~5% in seniors vs ~2% in young) together with a significant nominal reduction in dendritic cells (p = 0.045, FDR = 0.076; ~2% in seniors vs ~3% in young) (**Supplementary Table 24**). Some adaptive compartments showed a reduction in senior canines, most notably B cells (p = 3.010 x 10^-4^, FDR = 0.002; ~13% vs ~21% in young) and a trend toward fewer CD4^+^ T cells (p = 0.054, FDR = 0.076; ~39% in seniors vs ~45% in young), while CD8^+^ T cells increased significantly in seniors (p = 0.010, FDR = 0.036; ~19% in seniors vs 11% in young) (**Figure 2C**; **Supplementary Table 24**).

**Figure 3:**
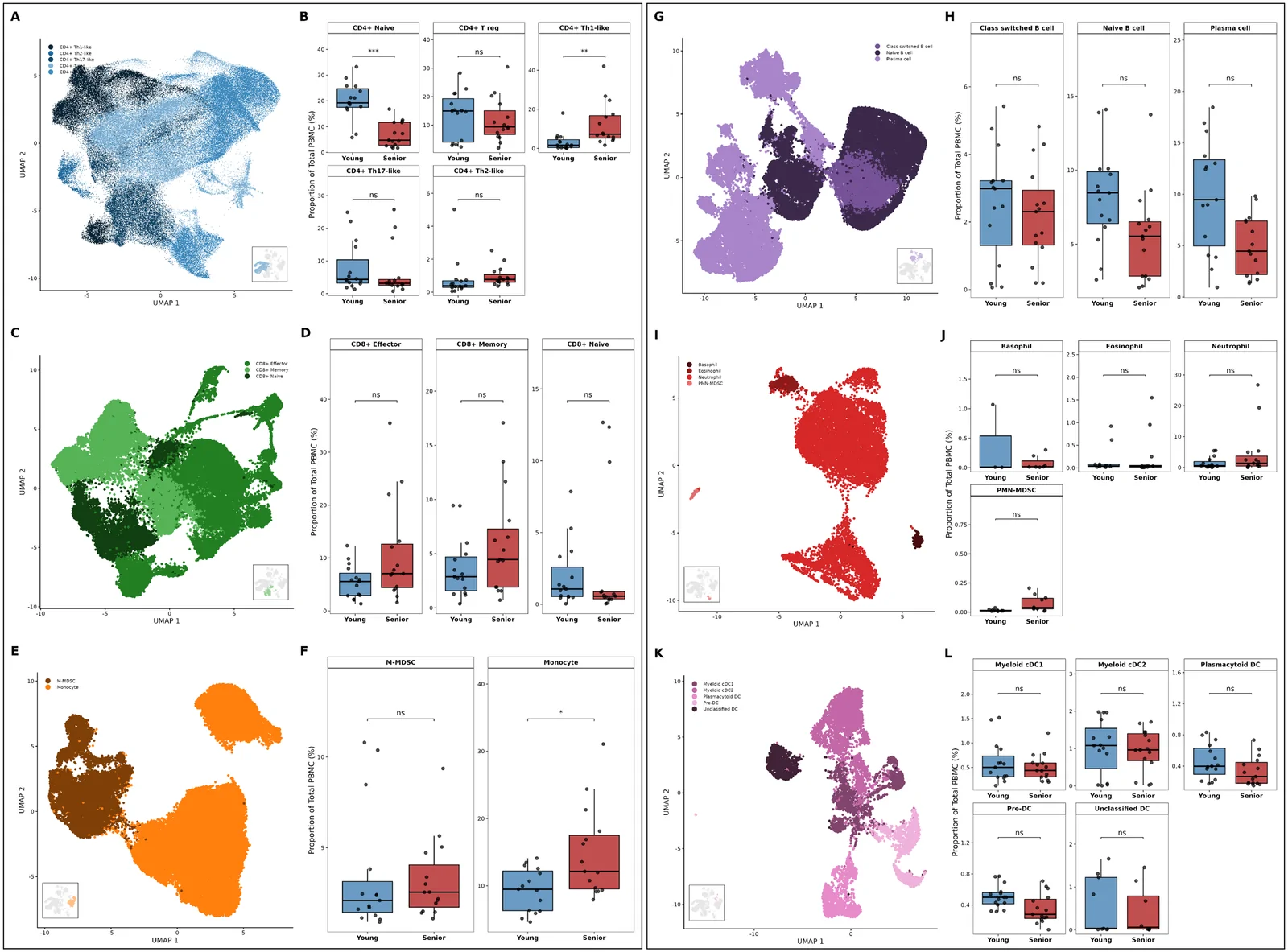
Sub-clustering analysis of major PBMC lineages. Detailed visualization and quantification of cellular subtypes within CD4+ T cells **(A, B)**, CD8+ T cells **(C, D)**, monocytes **(E, F)**, B cells **(G, H)**, granulocytes **(I, J)**, and dendritic cells **(K, L)**. Box plots represent the frequency of each subtype as a percentage of total PBMCs. Statistical significance was determined using propeller (speckle) with Benjamini–Hochberg FDR adjustment (ns: not significant, *FDR < 0.05, **FDR < 0.01, ***FDR < 0.001). Young n = 15, Senior n = 15.

To identify candidate contributors to these shifts, we examined lineage subclusters (**Figure 3**; **Supplementary Figure 1**, **Supplementary Table 25**). Neutrophil frequencies were increased in seniors, however not significantly (p = 0.194, FDR = 0.345; ~4.5% vs ~1.5% in young) (**Figures 3I, J**). In the dendritic cell lineage, senior canines showed a nominal reduction of the rare pre-dendritic cells (p = 0.014, FDR = 0.064; 0.3% vs 0.5% in young) (**Figures 3K, L**), whereas both cDC1 and cDC2 and other myeloid populations, including M-MDSC like, eosinophils, and basophils remained unchanged (**Figure 3**).

Adaptive immune remodeling was characterized by contraction of naïve compartments and selective enrichment of inflammatory phenotypes. The naïve CD4^+^ T-cell pool was substantially reduced in senior canines (20% vs. 7%, p = 4.529 x 10^-6^, FDR = 0.0001), accompanied by a significant expansion of CD4^+^ Th1 like cells (p = 1.498 × 10^-4^, FDR = 0.001;~12% vs 3% in young), whereas other helper populations including CD4^+^ regulatory T like cells (T reg like), and CD4^+^ Th17-like cells remained stable while CD4^+^ Th2-like cells were moderately perturbed (p = 0.055, FDR = 0.135) (**Figure 3B**; **Supplementary Table 25**). Consequently, the Th1/Th2 balance was elevated in senior canines (p = 0.030; log_2_FC = 1.6), consistent with a shift toward a more pro-inflammatory CD4^+^ T-cell profile inferred from transcriptional annotations (**Figure 3B**; **Supplementary Table 25**). Cycling T cells did not differ significantly between groups, while gamma-delta T cells were nearly depleted in senior canines (p = 1.952 × 10^-4^, FDR = 0.0013; ~0% vs 1% in young) (**Supplementary Figure 1**; **Supplementary Table 25**). The B-cell compartment showed nominal reductions in seniors for naïve B cells (p = 0.037, FDR = 0.117; ~6% vs ~8% in young) and plasma cells (p = 0.021, FDR = 0.082; ~5% vs ~10% in young) (**Figures 2G, H**; **Supplementary Table 25**).

Collectively, these compositional changes reveal a canonical immune-aging pattern in canines, consistent with expansion of selected myeloid and pro-inflammatory populations and depletion of naïve lymphocytes.

### Aging depletes cell-cycle & DNA repair pathways, activates inflammatory signaling in canine PBMCs

To determine if the observed changes in cellular composition were accompanied by canonical aging-related transcriptional changes, we performed pseudobulk differential expression analysis on the single-cell dataset. This identified 90 differentially expressed genes (DEGs) between senior and young dogs (**Figure 2D**; **Supplementary Table 2**). However, this global aggregation did not yield significant enrichment for Gene Ontology (GO) (24) terms or Kyoto Encyclopedia of Genes and Genomes (KEGG) (25) pathways when restricted to DEGs alone. To increase sensitivity and capture broader functional shifts, Gene Set Enrichment Analysis (GSEA) (26) was applied to all differentially expressed genes from the pseudobulk analysis.

GSEA identified 349 significant (FDR-corrected p-value < 0.05) GO Biological Process (BP) terms, of which 220 were upregulated in senior canines (normalized enrichment score, NES > 0) and 129 were downregulated (NES < 0) (**Supplementary Table 3**). In parallel, 78 KEGG pathways were identified as significant, 49 upregulated and 29 downregulated (**Supplementary Table 4**). Downregulated pathways and terms were dominated by ribosome, cell cycle, and DNA repair associated programs including ribosome biogenesis (GO:0042254; NES = -1.9), cell cycle checkpoint signaling (GO:0000075; NES = -1.7), cell cycle phase transition (GO:0044770; NES = -1.7), DNA replication (cfa03030 and GO:0006260; NES = -2.0 and -1.9), and base excision repair (cfa03410; NES = -1.9).

Conversely, upregulated pathways and terms showed an enrichment of the complement and coagulation cascades (cfa04610; NES = 2.0) and toll-like receptor signaling pathway (cfa04620; NES = 1.8). Additional immune effector programs, including Th1 and Th2 cell differentiation (cfa04658) and natural killer cell mediated cytotoxicity (cfa04650, GO:0042267) were also upregulated (NES = 1.7 for both) consistent with the increased CD4^+^ Th1-like and effector cell compartments observed at the compositional level. This global inflammatory shift was supported by enrichment of key “inflammaging” signaling hubs, including the NF-κB signaling pathway (cfa04064, NES = 1.7), TNF signaling pathway (cfa04668, NES = 2.0), and MAPK signaling pathway (cfa04010, NES = 1.7).

Together these results indicate that aging in canine PBMCs is associated with reduced expression of cell-cycle and DNA repair programs and heightened activation of inflammatory signaling pathways in circulating immune cells.

### Whole blood bulk transcriptomics reinforce inflammatory activation and cell-cycle depletion aging signatures

**Figure 4:**
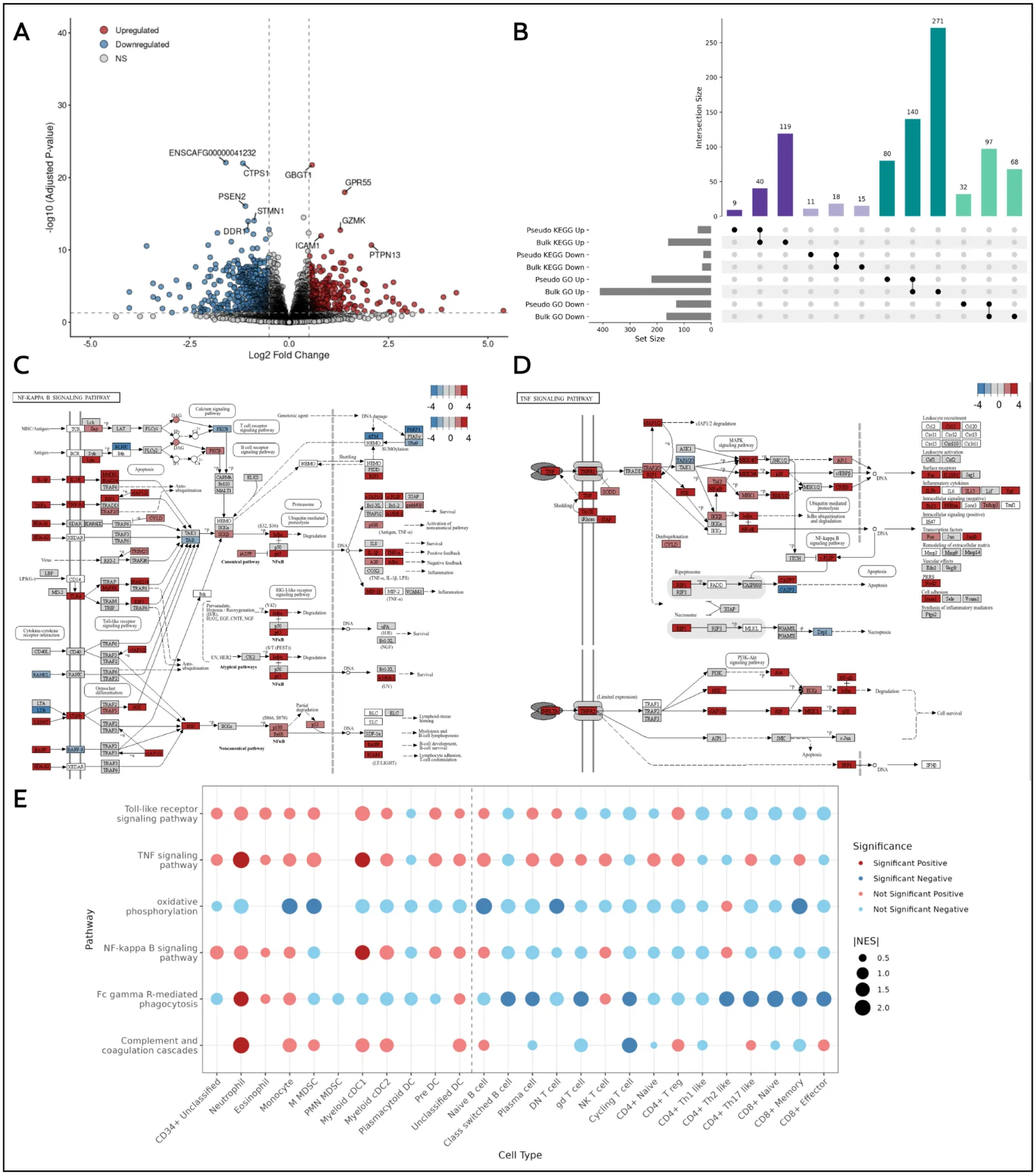
Bulk and single-cell transcriptomics reveal shared inflammatory signaling pathways. **(A)** Volcano plot displaying differentially expressed genes (DEGs) from whole blood bulk RNAseq analysis. Each dot represents a gene; red points indicate significantly upregulated genes in senior dogs, blue points indicate significantly downregulated genes in senior dogs, and grey points represent non-significant genes. Top DEGs are labeled with their gene symbols. **(B)** UpSet plot illustrating the intersection of significantly enriched Gene Ontology (GO) terms and KEGG pathways between the bulk RNA-seq overrepresentation analysis (ORA) and pseudobulk Gene Set Enrichment Analysis (GSEA). Vertical bars show the number of unique and shared pathways between upregulated (Up) and downregulated (Down) gene sets in both sequencing modalities. **(C, D)** KEGG pathway maps for the NF-κB signaling pathway **(C)** and TNF signaling pathway **(D)**. Genes colored in red are upregulated in senior dogs, while those in blue are downregulated in senior dogs in the differential expression analysis. **(E)** Dot plot summarizing GSEA across specific cell types. Rows represent selected inflammatory pathways, and columns represent annotated cell types. The size of each dot corresponds to the absolute Normalized Enrichment Score (|NES|), and the color indicates the significance and direction of regulation (Red: Significant Positive in senior dogs; Blue: Significant Negative in senior dogs; Faded colors: Not Significant; Left of dashed line: Innate cells, right of dashed line: adaptive).

Given the limited number of significant DEGs obtained from single-cell pseudobulk analysis, we next asked whether whole blood bulk transcriptomes from a larger cohort would recover the same aging-associated transcriptional programs. We profiled whole blood RNA from an expanded cohort of 39 canines (20 young and 19 senior). In contrast to the sparse nature of single-cell data, this analysis identified 3,265 significant differentially expressed genes (FDR-corrected p value < 0.05), with 1,636 upregulated and 1,629 downregulated in senior canines (**Supplementary Table 5**; **Figure 4A**). Of the 90 DEGs detected in the single-cell pseudobulk analysis, 87 were detected as differentially expressed in the bulk dataset. Among these, 51 (57%) were significant in the bulk dataset in the same direction of effect, an additional 30 showed concordant directional values but did not reach significance, and only 6 exhibited directional values in the opposite direction but did not reach significance (**Supplementary Table 6**). Overall, this pattern indicates broad directional concordance between the two platforms despite differences in sample size.

Using this larger DEG set, we performed functional overrepresentation analysis (ORA) to characterize pathways and terms associated with aging. In total, 411 GO BP terms were enriched among genes upregulated in seniors and 165 GO BP terms among downregulated genes (**Supplementary Tables 7**, **8**). KEGG pathway analysis identified 159 upregulated pathways and 33 downregulated pathways in the senior cohort (**Supplementary Tables 9**, **10**).

To directly compare the pathway-level signals between the single-cell and bulk analyses, we intersected pseudobulk GSEA results with bulk ORA (**Supplementary Table 11**; **Figure 4B**). For KEGG, 40 of 49 pathways enriched in seniors in pseudobulk GSEA (82%) were also significantly enriched among upregulated genes in bulk, and 18 of 29 downregulated pathways (62%) overlapped between analyses, with no pathways showing opposite directions of effect (i.e., upregulation in pseudobulk vs. downregulation in bulk, or vice versa) (**Figure 4B**). For GO BP terms, 140 of 220 upregulated terms (64%) and 97 of 129 downregulated terms (75%) were shared between pseudobulk GSEA and bulk ORA, again with no directionally discordant pathways (**Figure 4B**). The larger number of enriched terms unique to the bulk ORA analysis likely reflects the greater statistical power of the bulk dataset, whereas the high fraction of overlapping pathways indicates strong directional concordance between the two approaches. It is important to note that while the single-cell transcriptomics data were generated from isolated PBMCs, the bulk RNA-seq was performed on whole blood stabilized in PAXgene tubes. Consequently, the bulk dataset retains the massive population of circulating granulocytes (primarily neutrophils) that are intentionally depleted during standard PBMC isolation. The robust innate immune and inflammatory signaling signatures observed in the bulk RNA-seq dataset such as NF-κB and TNF signaling are therefore likely driven in large part by this whole-blood granulocyte fraction. This whole-blood signature provides a complementary, systemic view that reinforces the targeted myeloid expansion and inflammatory polarization observed in the single-cell PBMC data.

Consistent with pseudobulk GSEA results, downregulated genes were strongly enriched for pathways involved in cell-cycle regulation, DNA replication, and DNA repair, supporting a broad reduction in proliferative and genome maintenance programs in circulating immune cells (**Supplementary Tables 8**, **10**). Furthermore, upregulated genes showed significant enrichment of the NF-κB, TNF, MAPK signaling pathways (**Figures 4C, D**). This pro-inflammatory state was further reinforced from both analyses by the upregulation of the IL-17 signaling pathway (cfa04657, NES (pseudobulk) = 1.6, fold enrichment (FE; bulk) = 2.2) and the AGE-RAGE signaling pathway (cfa04933, NES (pseudobulk) = 1.6, FE (bulk) = 2.6).

Together, these bulk transcriptomic data provide an independent, higher-powered view of aging-associated gene expression changes in canine whole blood, reinforcing the combination of reduced cell-cycle and DNA repair programs with heightened inflammatory signaling that emerged from the pseudobulk single cell analysis.

### Single-cell resolution identifies candidate contributors to aging signatures across immune cells

To deconvolute the aggregate aging signature, we performed pathway analysis at the level of individual immune populations by applying a per-cell-type differential expression analysis coupled with GSEA. This analysis identified 286 unique GO BP terms showing significant enrichment across cell types (**Supplementary Table 12**). Of these, 113 terms were enriched in seniors (NES > 0) and 176 were depleted (NES < 0), with 3 terms exhibiting opposite directions of effect in different cell types. For KEGG, we identified 167 unique KEGG pathways with significant enrichment (**Supplementary Table 13**), including 46 pathways enriched in seniors (NES > 0) and 143 pathways depleted (NES < 0); 22 of the total pathways showed opposite directions across different lineages.

This per–cell-type analysis mapped the global inflammatory and cell-cycle signatures onto specific lineages. Consistent with the aggregate pseudobulk and bulk results, TNF signaling (cfa04668) was significantly enriched in neutrophils and myeloid cDC1 (NES > 1.8) (**Figures 4D, E**). Neutrophils and myeloid cDC1 also displayed a prominent inflammatory signature, with enrichment of inflammatory response (GO:0006954; NES > 1.7) and NF-κB signaling (cfa04064; NES > 1.5) (**Figures 4C, E**), indicating that these innate lineages are major contributors to the age-associated inflammatory pathways detected at the whole-PBMC level.

Pathway directionality was frequently divergent between innate and adaptive compartments, revealing how the aggregate PBMC signature masks lineage-specific differences. In total, 25 GO BP terms and KEGG pathways showed significant opposite direction of effect across different cell types (**Figure 4E**). For example, complement and coagulation cascades (cfa04610) were enriched in neutrophils (NES = 2.0) but depleted in cycling T cells (NES = −1.9). Similarly, platelet activation (cfa04620) was upregulated in neutrophils (NES = 1.5) yet downregulated in CD8^+^ effector and naive cells (NES < −1.7) (**Figure 4E**). FcγR–mediated phagocytosis (cfa04666) was enriched in neutrophils (NES = 1.8) but showed depletion across adaptive immune populations, including Th2-like, Th17-like, CD8^+^ effectors, and CD8+ naive cells (NES < −1.8) (**Figure 4E**).

Together, these results support an immune-aging phenotype specific to innate lineages alongside impaired or attenuated pathway activity in adaptive cells, rather than a uniform transcriptional shift across all immune compartments.

### Transcriptomics and metabolomics reveal extensive depletion of ether lipids and central metabolic intermediates

**Figure 5:**
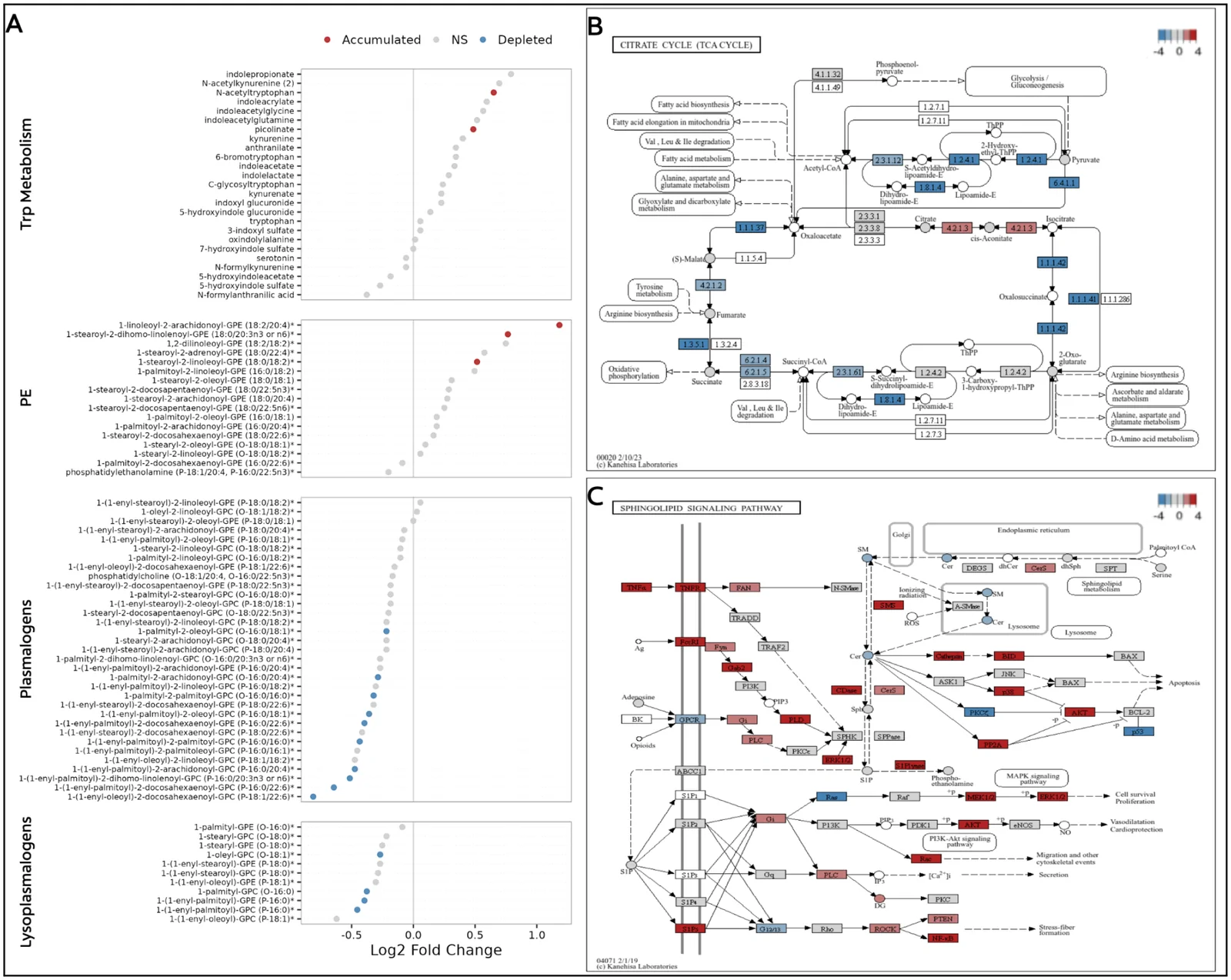
Transcriptomics and metabolomics reveal extensive depletion of ether lipids and dysregulation of central carbon metabolism. **(A)** Dot plots displaying the Log_2_FC of metabolites within key differentially enriched Metabolon sub-pathways. Red dots indicate significantly accumulated metabolites (FDR < 0.05) in senior canines, blue dots indicate significantly depleted metabolites, and gray dots represent non-significant trends. The plots highlight the accumulation of Tryptophan metabolites and Phosphatidylethanolamines (PE), contrasting with the widespread depletion of Plasmalogens and Lysoplasmalogens. **(B)** Integrated visualization of the Citrate (TCA) Cycle pathway (cfa00020). Rectangles represent differentially expressed genes (from bulk RNA-seq) and circles represent differentially abundant metabolites. Red indicates upregulation/accumulation in senior dogs and blue indicates downregulation/depletion in senior dogs. The map illustrates a bottleneck in bioenergetics characterized by the accumulation of Citrate and Pyruvate (red circles) concurrent with the broad downregulation of enzymatic machinery (blue rectangles). **(C)** Integrated visualization of the Sphingolipid Signaling Pathway (cfa04071). Following the same schema as **(B)**, the map depicts the transcriptional upregulation of signaling receptors and kinases (red rectangles) amidst complex shifts in lipid substrates, aligning with the observed remodeling of membrane composition and signaling responsiveness.

To characterize the systemic physiological shifts associated with aging, we performed untargeted serum metabolomics on all 50 canines. It is important to note that circulating metabolites reflect systemic physiology originating from multiple tissues, rather than direct intracellular immune-cell metabolism. We detected a total of 1,001 metabolites belonging to 105 Metabolon-designated sub-pathways and identified 73 differentially abundant metabolites (DAMs) between young and senior canines (FDR-corrected p value < 0.05) (**Supplementary Table 14**). Of these, 38 metabolites were accumulated in senior canines within 21 sub-pathways and 35 were depleted in a total of 15 sub-pathways. Sub-pathway enrichment analysis showed a significant (FDR-corrected p value < 0.05) accumulation in metabolites associated with Primary and Secondary bile acid metabolism (NES = 1.85 and 1.92) in seniors, whereas metabolites associated with plasmalogen (NES = -2.6), Long Chain Polyunsaturated Fatty Acid (n3 and n6) (NES = -2.3), sphingomyelins (NES = -1.9), lysoplasmalogen (NES = -2.1), Fatty Acid, Dihydroxy (NES = -1.87) and ceramides (NES = -2.0) were significantly depleted (**Supplementary Table 15**; **Figure 5A**), indicating broad shifts in ether and sphingolipid pools.

Functional pathway enrichment analysis identified 30 pathways enriched among accumulated metabolites (2 Reactome, 4 WikiPathways) and 4 enriched among depleted metabolites (all WikiPathways) (**Supplementary Tables 16**, **17**). In the transcriptomics data, we observed upregulation of lipid-related programs including response to lipid (GO:0033993, NES (pseudobulk) = 1.7, FE (bulk) = 2.1), sphingolipid signaling pathway (cfa04071, NES (pseudobulk) = 2.8, FE (bulk) = 2.8) and vesicle-mediated transport to the plasma membrane (GO:0098876, FE (bulk) = 2.5). Consistently, functional pathway analysis confirmed the depletion of the integrated sphingolipid metabolism pathway (WP4726). Interestingly, in the transcriptomics data, we observed a contrasting upregulation of lipid-related programs across the immune landscape, including response to lipid (GO:0033993, NES (pseudobulk) = 1.7, FE (bulk) = 2.1), sphingolipid signaling pathway (cfa04071, NES (pseudobulk) = 2.8, FE (bulk) = 2.8), and vesicle-mediated transport to the plasma membrane (GO:0098876, FE (bulk) = 2.5). Together, these findings indicate that the transcriptional upregulation of lipid signaling in immune cells may serve as a compensatory response to the systemic depletion of circulating membrane-associated lipids.

In addition to structural lipid depletion, senior canines demonstrated a marked reduction in circulating polyunsaturated fatty acids (PUFAs) and their bioactive signaling derivatives. MSEA revealed significant depletion of long-chain PUFAs (n3 and n6) (NES = -2.29) and dihydroxy fatty acids (NES = -1.87). This was further supported by the functional depletion of linoleic acid oxylipin metabolism (WP5137) and octadecanoid formation from linoleic acid (WP5324). Given that oxylipins and dihydroxy fatty acids act as critical lipid mediators in the resolution of inflammation and vascular homeostasis, their systemic depletion suggests a compromised capacity to maintain immune homeostasis and regulate inflammatory signaling with age.

While structural and signaling lipids were systemically depleted, the serum metabolome of senior canines was characterized by a broad accumulation of catabolic breakdown products and bile acids. MSEA identified a highly significant accumulation of both secondary (NES = 1.92) and primary bile acid metabolism (NES = 1.85), pointing toward age-related alterations in the hepatic-gut microbiome axis. Furthermore, functional enrichment highlighted an extensive accumulation of nucleotide and amino acid degradation products. Pyrimidine catabolism (R-HSA-73621) and broader nucleotide catabolism (R-HSA-8956319) were highly enriched in older subjects, indicative of increased cellular turnover, DNA damage, or senescence.

This accumulation of circulating catabolites occurred against a backdrop of widespread metabolic suppression at the cellular level. Transcriptomic analysis of the adaptive immune landscape indicated a broad suppression of central energy pathways, including the including the downregulation of the citrate (TCA) cycle (cfa00020; FE = 4.8; **Figure 5B**), aerobic respiration (GO:0009060; FE = 2.0), and oxidative phosphorylation (cfa00190; GO:0006119), most notably in plasma, CD8+ memory, and naive B cells (NES < -1.8). Furthermore, the systemic accumulation of amino acid breakdown products, including tryptophan metabolism (WP465) and phenylalanine catabolism (WP4156), was complemented by the transcriptional suppression of amino acid biosynthesis (cfa01230) across naive B cells, CD8+ effector, memory, and naive cells (NES < -1.6). Notably, neutrophils showed upregulation of the sphingolipid signaling pathway (cfa04071; $NES=1.7$; **Figure 5C**), reinforcing a divergence between innate and adaptive metabolic remodeling. Ultimately, the aging canine phenotype is defined by an adaptive immune system undergoing central energy suppression, coupled with a systemic environment depleted of essential lipid mediators and saturated with the byproducts of nucleotide and amino acid catabolism.

### Supervised integration of bulk transcriptomics and metabolomics identifies a potential molecular aging signature

**Figure 6:**
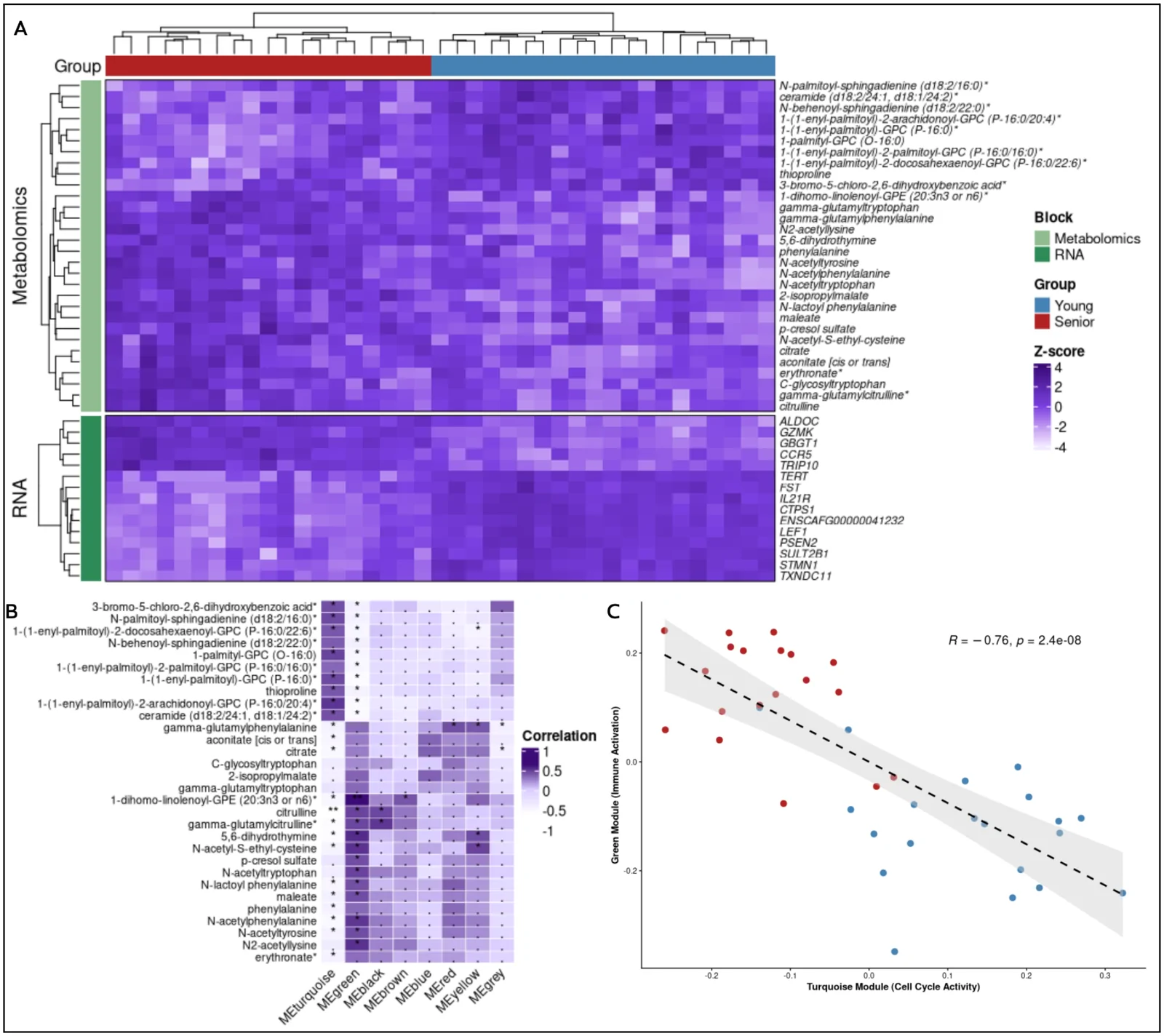
Integration of metabolomic and transcriptomics. **(A)** Clustered heatmap of gene and metabolite discriminators identified by DIABLO, demonstrating distinct molecular signatures by age group. **(B)** WGCNA heatmap showing correlations between gene co-expression modules and metabolites identified by DIABLO, *FDR < 0.05, **FDR < 0.01, “.” indicates no significance. **(C)** Correlation plot of the two dominant modules; samples cluster by age along a transition from Cell Cycle Activity (Turquoise) to Immune Activation (Green).

To determine if the observed shifts in lipid metabolism and bioenergetic capacity represent a coordinated systemic program, supervised integration of bulk transcriptomics and metabolomics was performed using DIABLO (Data Integration Analysis for Biomarker discovery using Latent Components) (27). This integration resulted in a molecular signature set of 15 genes and 30 metabolites (**Figure 6A**).

Senior canines showed accumulation of uremic solutes and amino acid catabolites (e.g., p-cresol sulfate, N-acetylphenylalanine, and N-lactoylphenylalanine) alongside the upregulation of key pro-inflammatory and senescent markers, including *CCR5* and *GZMK*. Conversely, young canines showed accumulation of structural ether lipids, specifically three plasmalogen species: 1-(1-enyl-palmitoyl)-2-arachidonoyl-GPC (P-16:0/20:4), 1-(1-enyl-palmitoyl)-2-docosahexaenoyl-GPC (P-16:0/22:6), and 1-(1-enyl-palmitoyl)-2-palmitoyl-GPC (P-16:0/16:0). In addition to two lysoplasmalogens: 1-(1-enyl-palmitoyl)-GPC (P-16:0) and 1-palmityl-GPC (O-16:0).

To contextualize these results, we utilized Weighted Gene Co-expression Network Analysis (WGCNA) (28) to generate functional modules from bulk transcriptomics data and correlate them with this focused set of discriminant metabolites. Correlation of the module eigengenes revealed an inverse relationship between the Turquoise module, and mainly the Green module (**Figure 6B**). The Turquoise module contained 1208 genes and was positively correlated with all the plasmalogens, lysoplasmalogen, and sphingolipid species (**Figure 6B**). ORA analysis showed an enrichment in cell cycle activity genes (GO:0007049, cfa04110) (**Supplementary Tables 26**, **27**). The Green module contained 593 genes and was positively correlated with amino acid metabolism and uremic toxins.These genes showed enrichment in varied immune processes including immune response (GO:0006955), immune effector process (GO:0002252), and natural killer cell mediated cytotoxicity (cfa04650) (**Supplementary Tables 28**, **29**).

We observed a highly significant inverse correlation between the Turquoise (Cell Cycle) and Green (Immune Activation) module eigengenes (R = -0.76, p = 2.4 x 10^-8^; **Figure 6C**). This linear relationship indicates that the Turquoise module eigengene accounts for a proportions of the variance in the Green module, a strong correlation that is consistent with, but does not establish, a metabolic–transcriptomic trade-off in which loss of structural lipid maintenance may co-occur with escalating systemic inflammation during canine aging; functional studies are needed to test this.

### Transcriptomics and clinical biomarkers associate downregulated genes with age related traits

**Figure 7:**
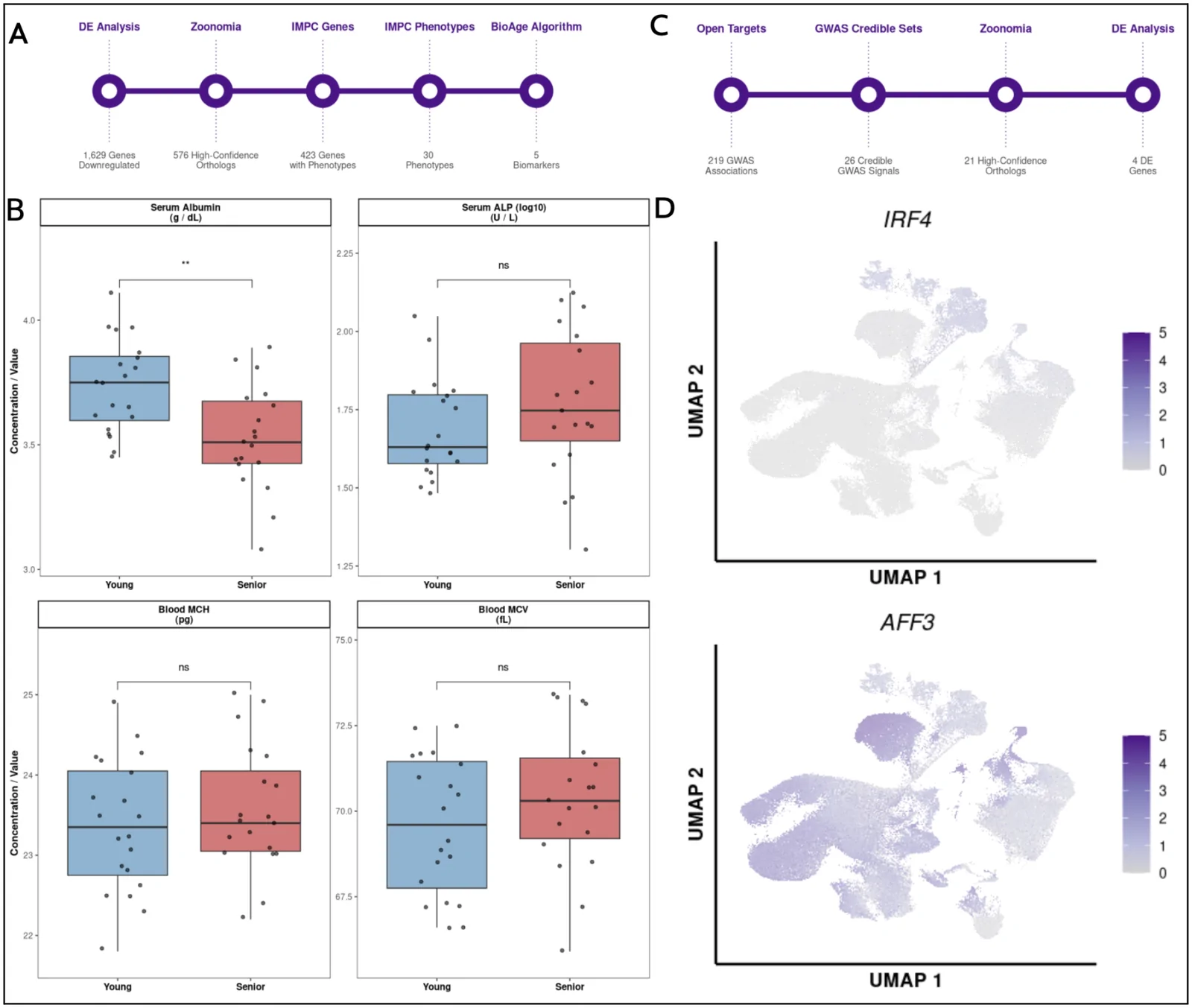
Integrated cross-species analysis prioritizes genes with potential roles in canine aging. **(A)** Murine phenotypes-driven pipeline: 1,629 genes downregulated in senior canines were filtered via Zoonomia orthology, IMPC phenotypes, and BioAge algorithm to identify candidates. **(B)** Physiological validation. Box plots comparing blood biomarkers (Albumin, ALP, MCH, MCV) in Young (blue) vs. Senior (red) dogs, **P < 0.01. **(C)** Human GWAS-driven pipeline. Aging GWAS hits were filtered for credibility, canine orthology, and differential expression. **(D)** UMAPs showing single-cell expression of prioritized candidates (*IRF4, AFF3*).

In order to connect age-associated transcriptional changes to organismal phenotypes, loss-of-function data from IMPC was leveraged (**Figure 7A**). The focus was directed toward genes downregulated with age in canines, for the reason that concordance between reduced gene expression in seniors and phenotypes observed in murine knockouts would be consistent with age-related functional decline. Downregulated canine genes were mapped to high-confidence murine orthologs using Zoonomia¹^0^ (see Methods), yielding 576 (35%) high-confidence orthologs out of 1,629 downregulated genes. Importantly, because all expression changes were measured in PBMCs, this framework specifically associates transcriptional aging in circulating immune cells to organismal phenotypes, providing an immune-centric view into aging biology.

Cross-referencing these orthologs with IMPC phenotype annotations identified multiple traits relevant to aging (**Supplementary Table 18**). For example, 12 genes were associated with decreased lymphocyte cell number. Consistent with this, senior canines in our cohort showed lower lymphocyte counts than young (**Figure 2C**). Additional IMPC phenotypes linked to downregulated orthologs included increased total body fat, impaired glucose tolerance, and decrease in bone mineral density, aligning with well established systemic aging-related traits in mice and humans.

As an example application in the canine health and longevity space, the gene–phenotype pairs were intersected with a recently proposed panel of 10 clinical measurements that predict biological age in canines (BioAge) ^9^. Five of the 10 biomarkers: serum albumin, alkaline phosphatase (ALP), circulating serum glucose, mean corpuscular hemoglobin (MCH), and mean corpuscular volume (MCV), were represented among the significant IMPC gene–phenotype associations (**Supplementary Table 19**). Although these biomarkers are not immune-specific, they are tightly linked to systemic inflammatory and metabolic states that modulate immune function, making them relevant readouts in the context of immune aging. For serum albumin, 14 downregulated genes were associated in IMPC with decreased levels, and senior canines in this cohort showed significantly lower serum albumin compared to young canines with concordant direction across IMPC, our data, and the BioAge model (p = 0.001; **Figure 7B**). Similarly, and also concordant across all three resources, 28 downregulated genes were associated with increased circulating alkaline phosphatase (ALP) levels in IMPC. ALP was not different between seniors and young canines in this cohort (**Figure 7B**). MCH and MCV showed an increase concordant with the IMPC phenotypes but differed in the BioAge model (**Figure 7B**), which reported age-related decreases in these indices. A full summary of all the clinical and serum biomarkers is reported in **Supplementary Table 30**.

### GWAS guided interpretation of transcriptomics prioritizes genes with a potential role in aging

To prioritize genes with convergent evidence for involvement in aging, we curated 39 candidates from three canine longevity and lifespan GWAS studies (**Supplementary Table 20**). Eleven of these 39 genes (28%) were significantly differentially expressed with age in our cohort (downregulated: *AP1B1*, *PELP1*, *THOC5*, upregulated: *B3GALNT1*, *BANK1*, *BCL6*, *EPAS1*, *MTMR3*, *NLRP1*, *PARP14*, *PLA2G4A*). Four of the 11 genes showed broad expression across multiple PBMC lineages and lacked clear cell-type specificity (**Supplementary Figure 3**). In contrast, several genes exhibited strong restriction to specific cell-type subsets: for example, *BANK1* expression was concentrated in naive B cells, which are significantly decreased in senior canines (**Supplementary Figure 3**) and *BCL6* expression was enriched in eosinophils, whose overall abundance showed no differences with age (**Supplementary Figure 3**). The remaining genes, including *NLRP1* and *PLA2G4A* showed specificity to cell types expanded in senior canines such as monocytes and PMN-MDSC like (**Supplementary Figure 3**). This suggests that some canine GWAS candidates may contribute to aging through changes in cell type abundance in addition to cell-intrinsic regulation, which has been previously shown in human studies (29).

Given the limited number and size of canine GWAS, we next leveraged human aging GWAS to extend this analysis (**Figure 7C**). We queried the Open Targets Platform ¹¹ for genes associated with the Experimental Factor Ontology (EFO) term (Aging; EFO_0002507) and retained those with a high GWASCredibleSet score (see Methods). This yielded 26 human candidates, of which 21 had high-confidence human–canine orthologs in Zoonomia ¹^0^ (**Supplementary Table 21**). Intersecting these 21 orthologs with differentially expressed genes from our cohort produced a set of four genes: *IRF4*, *AFF3*, *RPN1*, and *TMEM263*, all significantly downregulated in senior dogs.

Among these, *IRF4* (Interferon Regulatory Factor 4) emerged as a particularly compelling candidate. *IRF4* knockout was associated with decreased lymphocyte cell numbers in IMPC and exhibited strong cell-type specificity in PBMCs, with expression almost exclusively in plasma cells, which were reduced in abundance in senior canines (p = 0.016; **Figures 3G, H**, **7D**). In human aging GWAS, *IRF4* carries a high locus-to-gene (L2G) score (~0.95) and strong eQTL colocalization support (CLPP ≈ 0.94), and its knockout was associated with a decrease in lymphocyte numbers in IMPC, strengthening *IRF4* as a cross-species candidate with potential role in aging-related phenotypes. Another gene, *AFF3* (ALF transcription elongation factor 3), had a high L2G score (~0.87), and its knockout was associated, via IMPC, with increased alkaline phosphatase and decreased serum albumin, concordant with both IMPC phenotypes and the BioAge model (**Figure 7D**; **Supplementary Table 19**).

## Discussion

Aging in canines has largely been examined through isolated datasets, limiting the ability to connect immune, metabolic, and genetic changes into a coherent biological framework. Here, by combining what is, to our knowledge, the largest canine PBMC single-cell atlas to date (**Supplementary Table 1**) (64–70) with bulk transcriptomics, serum metabolomics, clinical biomarkers, and integrating them with external genetic and functional resources (IMPC, Zoonomia, Open Targets), we describe a coordinated, multi-layered program of immune and metabolic remodeling with age in canines.

Our single-cell data revealed a pattern consistent with inflammaging in the PBMC fraction of senior canines. Bulk and pseudobulk transcriptomics provided complementary evidence, with strong upregulation of innate immune pathways and marked downregulation of cell-cycle regulators, DNA replication factors, and DNA repair genes, consistent with reduced proliferative and repair programs in circulating immune cells (30). Because our bulk transcriptomics were derived from whole blood rather than isolated PBMCs, this dataset captured the full circulating granulocyte pool. The strong upregulation of innate immune pathways in the bulk data likely reflects this abundant neutrophil population, providing a systemic whole-blood context that reinforces the inflammaging phenotype observed in the single-cell atlas. While myeloid expansion was not uniform, indicating a targeted bias toward specific inflammatory and suppressor phenotypes, we note that granulocyte proportions derived from PBMC single-cell data must be interpreted cautiously, as they primarily reflect low-density fractions rather than the total circulating granulocyte pool. Per–cell-type pathway analysis further deconvoluted these aggregate signatures, localizing TNF signaling and NF-κB signaling, while multiple adaptive subsets showed depletion of cell-cycle, oxidative phosphorylation, and receptor-mediated pathways. In parallel, the elevation of the Th1/Th2 balance in senior canines reflected a systemic shift toward cell-mediated cytotoxicity over humoral support, a hallmark of immune aging in other species (31). Together, these compositional and transcriptional features are consistent with an aged canine immune system that is ‘primed’ for inflammatory reactivity rather than adaptive diversity, which are features of immunosenescence and inflammaging.

In aged dogs there was an apparent decrease in transcriptional signatures of energy production within immune cells, alongside systemic (serum) signatures of disrupted lipid antioxidants and cell survival/death pathways. Downregulation of TCA cycle and oxidative phosphorylation transcriptional pathways was accompanied by accumulation of TCA intermediates in the serum of senior canines. While this is consistent with reduced utilization of these metabolites by immune cells, these circulating metabolites reflect systemic physiology, and changes in their production or handling by other tissues cannot be excluded. Further, the widespread systemic depletion of plasmalogens and sphingolipids may reflect impaired membrane-lipid–dependent signaling with age, although serum measurements cannot localize this specifically to immune cells. Plasmalogens are critical endogenous sacrificial antioxidants that protect polyunsaturated fatty acids from oxidation to stabilize membranes (32). Their extensive loss suggests a compromised defense against oxidative stress, a key driver of inflammaging (33). Notably, core peroxisomal plasmalogen biosynthesis genes (*GNPAT*, *AGPS*, *FAR1*, *PEX7*) responsible for ether-linked lipid synthesis and breakdown, were not changed, whereas *PEX5*, a key receptor for transport of antioxidant catalase into peroxisomes was downregulated. *PEX5* and catalase have been shown to be oxidative stress sensors, where the defense mechanism is keeping catalase in the cytosol (34). Ceramides and sphingomyelins were also broadly decreased. Sphingolipids have been previously implicated in human aging (35), supporting a reorganization of cell signalling-associated lipid metabolism in aged canines. Together, ceramides and sphingomyelins act as a cellular rheostat for proliferation and cell death (36). Sphingomyelins play a role in the structural regulation of cell membrane lipid microdomains to promote survival, adhesion and proliferation. In contrast, ceramides lack a phosphocholine headgroup and serve largely as small molecule messengers promoting autophagy, apoptosis and senescence. Sphingosine-1-phosphate, a small molecule that regulates trafficking of immunocytes in spleen, thymus and lymph tissue (37), was also decreased in aged dogs. Receptors for sphingosine-1-phosphate are targets of immunosuppressive therapies (38), and a decrease in this messenger with age may indicate a decreased immunocompetence in senior dogs.

Multi-omic integration nominated a focused set of candidate genes with convergent evidence for involvement in aging, supported by our data, canine or human GWAS associations, and high-confidence cross-species orthology. One example is *PLA2G4A*, a cytosolic phospholipase A2, that hydrolyzes membrane phospholipids, including plasmalogens, to produce lysoplasmalogens and downstream arachidonic acid species implicated in inflammatory signaling (39). In our dataset, *PLA2G4A* was significantly upregulated in senior dogs and showed highest expression in rare myeloid populations (basophils and PMN-MDSC like). Together with its enzymatic role at the interface of membrane remodeling and eicosanoid production, the identification of a canine lifespan GWAS signal in *PLA2G4A* intronic regions (21), nominates this pathway as a candidate contributor to age-associated lipid and inflammatory changes that warrants direct functional validation. In combination with *PEX5* downregulation and plasmalogen depletion, *PLA2G4A* emerges as a plausible contributor to immunometabolic aging in canines, warranting direct functional follow-ups in the future.

## Limitations

Despite the strength of our multiomic approach, several limitations should be noted. First, our study is cross-sectional and conducted in a single breed (beagles). Furthermore, the study utilizes an extreme-phenotype design comparing distinct young and senior groups with a large middle-age gap. While this maximizes statistical power to detect broad age-related differences, it precludes the analysis of aging as a continuous variable and limits our ability to infer nonlinear temporal dynamics or generalize to broader canine populations. Second, all molecular measurements were made in PBMCs and serum; tissue-resident immune cells, bone marrow, and parenchymal organs were not assayed; therefore systemic traits inferred from IMPC may involve tissues beyond blood. It is important to note that while serum metabolites offer systemic insights, they should be interpreted with caveats regarding cellular metabolism, as circulating TCA intermediates may not mirror the intracellular dynamics of specific immune subsets. Also, because our single-cell transcriptomics were generated from isolated PBMCs, the dataset inherently underrepresents total circulating granulocytes, meaning findings related to neutrophils primarily reflect subpopulations that copurify with mononuclear cells. The per-animal single-cell granulocyte proportion correlated only weakly with the CBC neutrophil measure (Spearman ρ = 0.22, p = 0.23, **Supplementary Table 31**). In addition, the established links between one-carbon metabolism and age-related changes in DNA methylation ²¹, point to an epigenetic component of canine immune aging that was not examined in this study. Third, in our murine phenotype-guided analysis, age-related downregulation in canines represents a partial reduction in gene expression that may not fully recapitulate the binary phenotypes of complete genetic nulls. Finally, our human GWAS comparison assumes that the gene implicated by locus-to-gene scoring is the true causal gene and that its canine ortholog retains a conserved functional role in aging.

## Summary

In summary, this work establishes a reference for canine aging biology and provides a scalable framework for future multi-omic studies in companion animals. Our study outlines an immunometabolic view of canine aging with specific cellular and molecular changes in circulating immune cells (PBMCs and whole blood), serum metabolites, and clinical biomarkers. In addition, we prioritize genes with potential roles in canine aging for future functional validation. The underlying multi-omic resource can be used to address diverse questions in canine immunology, metabolism, and comparative aging. Ultimately, leveraging such integrative resources may help guide the development and testing of strategies aimed at improving the health and extending the lifespan of companion animals.

## Methods

### Study cohort

The study protocol was reviewed and approved by the Institutional Animal Care and Use Committee; Hill’s Pet Nutrition, Inc., Topeka, KS, USA and complied with the guides for the care and use of laboratory animals from the National Institutes of Health, US National Research Council and US Public Health Service. Age, breed, sex, and environmental survey information were reported by animal care technicians. We selected a total of 50 canines, 25 young with an average age of 3.9 ± 0.6 years and 25 senior canines with an average age of 12.1 ± 1.4 for untargeted serum metabolomics profiling. All dogs were clinically healthy adults, confirmed by veterinary examination and routine clinical screening (complete blood count and serum chemistry), with no clinically significant abnormalities at the time of sampling. For whole blood bulk RNAseq data, we studied a subset of 39 animals (20 young and 19 seniors) increasing the average age of seniors to 12.2 ± 1.2 years. We further reduced the number of canines selected for single-cell RNAseq to a subset of 30 which were divided into two equal groups: young canines with an average age of 3.3 ± 0.5 years, and senior canines with an average age of 11.4 ± 1.3 years. Sex was recorded for all animals and was balanced across age groups. Sex was included as a covariate in the bulk RNA-seq model; because sex was not associated with age group, it could not confound the age effect and was not additionally adjusted for in the single-cell and metabolomics analyses. All animals were maintained on the same complete and balanced adult maintenance diet throughout the study. All 30 single-cell samples were distributed across the same five Parse sub-libraries and sequenced on a single S4 flow cell; because differential expression was computed on per-animal pseudobulk profiles, sub-library-level technical variation is averaged within each sample and was not modeled as a separate batch term. Bulk RNA-seq libraries were sequenced on a single flow cell, and metabolomics data were batch-normalized by the vendor (Metabolon). All information is reported in **Supplementary Table 23**.

### Sample handling

Blood was collected in PAXgene tubes, maintained in a vertical orientation to prevent additive backflow while filling exactly to the 2.5mL mark and gently inverted 8–10 times to ensure RNA quality, then incubated upright at room temperature for a consistent duration (minimum 2 hours) to maintain data integrity. Finally, tubes were frozen upright in a wire rack at -20 °C for 24 hours before transferring to long-term -70 °C storage.

### PBMC isolation

4mL of venous whole blood was collected into a mononuclear cell preparation tube containing buffered sodium citrate (BD Biosciences). PBMC were then processed as described in the manufacturer’s protocol as follows. Blood tubes were centrifuged for 30 minutes at room temperature at 1800 relative centrifugal force (RCF). The mononuclear cell layer was collected and subsequently washed twice with Phosphate Buffered Saline (PBS). PBMC were then resuspended in a freeze media containing 10% DMSO (Biolife Solutions), stored at -80°C in a Mr. FrostyTM freezing container (Thermo Scientific) filled with 100% isopropyl alcohol for 24 hours until shipped on dry ice for further processing.

### Serum isolation

A minimum of 3.5 mL whole blood is collected via venipuncture into a vacutainer serum separator tube (SST) tube (Becton Dickinson). The blood is allowed to clot for 30 minutes before it is centrifuged at 2,000xG for 10 minutes. Serum is then placed into a 1.8mL cryovial and stored at -80°C until further processing.

### Single-cell RNA library preparation and sequencing

We performed single-cell RNA sequencing of PBMCs from 30 canine blood samples using Parse Biosciences (Seattle, WA, USA) Evercode^®^ Whole Transcriptome Mega Kit and Illumina^®^ (San Diego, CA, USA) sequencing technologies. Eremid (Kannapolis, NC, USA) performed sample QC, cell fixation, barcoding, library preparation, sublibrary generation, pooling, and sequencing on the NovaSeq6000 system.

Cell Fixation and QC: The PBMC samples were thawed, counted, and assessed for viability using Trypan Blue and the Countess II automated cell counter. Two counts were performed for each sample. A total of 30 samples met QC requirements for cell fixation. After cells were fixed, samples were counted, aliquoted, placed in a Mr. Frosty, and stored at -80C. An aliquot (10ul) was made to count cells and account for any freeze/thaw changes before barcoding.

Barcoding and Library Preparation: Fixed cells were thawed and counted before barcoding. All 30 samples plus 1 Parse internal control were loaded onto barcoding plate 1 and underwent three rounds of barcoding and pooling before ultimately being counted and split into 5 sub-libraries and were quantified using Qubit and Bioanalyzer.

Sequencing: Sublibraries 1–5 were combined at equimolar ratio and loaded, along with (Calculated 7% PhiX but the run did not reflect this) into one S4 flow cell for clustering. The sequencing was performed using a 200-cycle run on the NovaSeq6000 instrument with the following Evercode Whole Transcriptome v3 specifications.

### Single-cell RNAseq analysis

Preprocessing: Sequencing output was demultiplexed into 5 sub-libraries containing 30 samples each. Data preprocessing to produce the expression matrix needed for downstream analysis was conducted using ParseBiosciences-Pipeline (v1.2.1). In short, we created a Parse compatible index for CanFam3.1 using ‘mkref’ mode. Next, we performed quality control using FastQC (v0.12.0), and alignment using STAR (v2.7.11a) (40). Summary statistics were generated for each individual sub-library in accordance with Parse recommendation using ‘all’ mode. The final step was performed using “combine” mode to combine all the sub-libraries and create the metadata and expression matrix required for downstream analysis.

Downstream analyses were conducted in R (v4.4) using Seurat (v5.3) (41) with support from other R packages. First, to help eliminate very rare/noisy genes, we removed genes expressed in fewer than 10 cells using ‘min.cells = 10’. To filter poor quality cells, we filtered cells with fewer than 250 detected genes and cells with higher than 5% mitochondrial gene expression using the pattern ‘^MT’. The preprocessed Seurat object was normalized with SCTransform (v2) to model and mitigate technical noise and regressing mitochondrial effects. Highly variable features were identified from the SCTransform-normalized data, and principal component analysis was performed. We opted to select the first 30 PCs based on scree plot to build a shared nearest neighbor graph, these PCs were not correlated with ‘nCount’ or ‘mt.percent’. Global clustering was conducted at a resolution of 1.6, followed by UMAP for two-dimensional visualization. We obtained 58 clusters, with the largest cluster containing 21,448 cells and the smallest containing 83 cells. We identified cluster markers using ‘FindAllMarkers’ (only.pos = TRUE). To assign cell identities, cluster markers were matched to a curated reference of canine immune cell markers (15). First, we assigned each cell to a major lineage: CD8^+^ T, CD4^+^ T, B cell, granulocyte, monocyte, dendritic, and miscellaneous. Next, for each major lineage we performed sub-clustering using 10–20 PCs and a resolution of 0.1-0.6 to determine the subtypes.

Cell-type composition comparisons: To determine if cell type abundance differed significantly between age groups, account for the compositional nature of single-cell data, and improve statistical power through variance shrinkage, we performed cell-type composition comparisons using the ‘propeller’ function implemented in the speckle (v1.6.0) R package (42). Reported propeller p-values were adjusted for multiple testing across cell types using the Benjamini–Hochberg false discovery rate (FDR); FDR < 0.05 was considered significant. Cell-type proportions were transformed using the logit transformation to normalize the data range. A robust linear model was fitted (robust = TRUE) with an empirical Bayes step (trend = TRUE) to estimate the mean-variance relationship and smooth standard errors in accordance with package recommendations.

Th1/Th2 balance quantification: We quantified Th1/Th2 balance per canine by aggregating single-cell annotations to counts of CD4^+^ Th1-like and CD4^+^ Th2-like cells for each sample, then computing a per-sample ratio using a small pseudocount to avoid division by zero (Th1 + 0.5)/(Th2 + 0.5) and log_2_-transforming the ratio. Group differences between young and old dogs were tested using a linear model on the log_2_ ratio (log_2_(Th1/Th2) ~ condition), and effect sizes were reported as log_2_ of model coefficient.

Differential expression analysis: Differential expression was performed using both a global pseudobulk and per-cell type strategy. In global pseudobulk, for each sample–condition combination, raw RNA counts were aggregated with Seurat’s AggregateExpression (assay = RNA, sum of counts), yielding integer pseudobulk profiles per group. Identities were set to the concatenated cell type and condition, and differential expression between young and senior canines was tested with DESeq2 (v1.46.0) via Seurat’s ‘FindMarkers’ (min.pct = 0), allowing DESeq2 to handle library-size normalization and dispersion estimation on the count data. Multiple testing correction was applied using the Benjamini–Hochberg procedure (BH/FDR), and significance was summarized at FDR < 0.05. To identify cell-type-specific changes associated with aging, we performed pseudobulk differential expression analysis. Single-cell gene counts were aggregated to the sample level separately for each cell type. Differential expression testing was performed independently for each cell type using DESeq2 (v1.46.0). We applied strict inclusion criteria: only cell types with at least 5 biological replicates per condition were tested. Furthermore, genes were filtered to retain only those with ≥10 counts in at least 3 samples. The resulting gene lists represent condition-specific changes within specific cell populations.

GSEA: For both strategies, genes were ranked by their average Log_2_FC. This ranked list was used to perform GSEA using ‘gseGO’ and ‘gseKEGG’ functions via the clusterProfiler (v4.16.0) R package leveraging Org.Cf.eg.db (v3.21.0). Pathways with a Benjamini-Hochberg adjusted p-value < 0.05 were considered significantly enriched.

### Bulk RNA library preparation and sequencing

RNA Extraction and QC: A total of 39 whole blood RNA extractions were performed using Qiagen’s PAXgene Blood RNA Kit IVD, following manufacturer’s instructions. The RNA samples were quantified fluorometrically using Qubit^®^ 3.0 (Thermo Fisher Scientific) and sample integrity was assessed using Bioanalyzer (Agilent^®^).

Library Preparation: RNA-Seq libraries were generated using the Universal Plus mRNA-Seq kit (Tecan^®^), following manufacturer’s guidelines, including poly(A) selection, fragmentation, cDNA synthesis, end repair, adaptor ligation, and amplification. Final libraries were assessed using qPCR (Bio-Rad^®^) and Bioanalyzer (Agilent^®^) prior to equimolar pooling. All libraries passed QC with an average peak size of 455 bp. Optimization qPCR protocol was followed to determine amplification cycles for each sample (**Supplementary Table 22**).

Sequencing: A total of 39 libraries were combined in equimolar concentration and loaded, along with 1% PhiX, into an SP flow cell for clustering. The sequencing was performed using a 218-cycle run on the NovaSeq 6000 instrument to generate 100 bp paired-end reads.

### Bulk RNAseq analysis

Quality control was performed using FastQC (v0.12.1). The samples were aligned using the splice aware aligner HISAT2 (v2.2.1) (43) with ensembl CanFam3.1 generated via ‘hisat-build’ function. Assembly was completed using StringTie (v3.0.0) (44). Input for downstream differential expression analysis was generated using ‘prepDE3.py’ within StringTie. Differential expression analysis was performed using DESeq2 (v1.46.0) in R (v4.5.1) correcting for sex. ORA was performed using ‘enrichGO’ and ‘enrichKEGG’ functions within clusterProfiler (v4.16) leveraging Org.Cf.eg.db (v3.21.0).

### Untargeted metabolomics

Untargeted metabolomics was performed by Metabolon (Morrisville, NC, USA).

#### Sample preparation

Samples were prepared using the automated MicroLab STAR^®^ system (Hamilton Company). Several recovery standards were added prior to the first step in the extraction process for QC purposes. To remove protein, dissociate small molecules bound to protein or trapped in the precipitated protein matrix, and to recover chemically diverse metabolites, proteins were precipitated with methanol under vigorous shaking for 2 min (Glen Mills GenoGrinder 2000) followed by centrifugation. The resulting extract was divided into multiple fractions: two for analysis by two separate reverse phase (RP)/UPLC-MS/MS methods with positive ion mode electrospray ionization (ESI), one for analysis by RP/UPLC-MS/MS with negative ion mode ESI, one for analysis by HILIC/UPLC-MS/MS with negative ion mode ESI, while the remaining fractions were reserved for backup. Samples were placed briefly on a TurboVap^®^ (Zymark) to remove the organic solvent. The sample extracts were stored overnight under nitrogen before preparation for analysis.

#### QA/QC

Several types of controls were analyzed in concert with the experimental samples: a pooled matrix sample generated by taking a small volume of each experimental sample (or alternatively, use of a pool of well-characterized human plasma) served as a technical replicate throughout the data set; extracted water samples served as process blanks; and a cocktail of QC standards that were carefully chosen not to interfere with the measurement of endogenous compounds were spiked into every analyzed sample, allowed instrument performance monitoring and aided chromatographic alignment. Instrument variability was determined by calculating the median relative standard deviation (RSD) for the standards that were added to each sample prior to injection into the mass spectrometers. Overall process variability was determined by calculating the median RSD for all endogenous metabolites (i.e., non-instrument standards) present in 100% of the pooled matrix samples. Experimental samples were randomized across the platform run with QC samples spaced evenly among the injections.

#### Ultrahigh performance liquid chromatography-tandem mass spectroscopy

All methods utilized a Waters ACQUITY ultra-performance liquid chromatography (UPLC) and a Thermo Scientific Q-Exactive high resolution/accurate mass spectrometer interfaced with a heated electrospray ionization (HESI-II) source and Orbitrap mass analyzer operated at 35,000 mass resolution (45). The dried sample extracts were then reconstituted in solvents compatible with each of the four methods. Each reconstitution solvent contained a series of standards at fixed concentrations to ensure injection and chromatographic consistency. One aliquot was analyzed using acidic positive ion conditions, chromatographically optimized for more hydrophilic compounds (PosEarly). In this method, the extract was gradient eluted from a C18 column (Waters UPLC BEH C18-2.1x100 mm, 1.7 µm) using water and methanol, containing 0.05% perfluoropentanoic acid (PFPA) and 0.1% formic acid (FA). Another aliquot was also analyzed using acidic positive ion conditions, however it was chromatographically optimized for more hydrophobic compounds (PosLate). In this method, the extract was gradient eluted from the same aforementioned C18 column using methanol, acetonitrile, water, 0.05% PFPA and 0.01% FA and was operated at an overall higher organic content. Another aliquot was analyzed using basic negative ion optimized conditions using a separate dedicated C18 column (Neg). The basic extracts were gradient eluted from the column using methanol and water, however with 6.5mM Ammonium Bicarbonate at pH 8. The fourth aliquot was analyzed via negative ionization following elution from a HILIC column (Waters UPLC BEH Amide 2.1x150 mm, 1.7 µm) using a gradient consisting of water and acetonitrile with 10mM Ammonium Formate, pH 10.8 (HILIC). The MS analysis alternated between MS and data-dependent MSn scans using dynamic exclusion. The scan range varied slightly between methods but covered 70–1000 m/z.

### Untargeted metabolomics raw data analysis

#### Data extraction and compound identification

Raw data was extracted, peak-identified and QC processed using a combination of Metabolon developed software services (applications). Each of these services perform a specific task independently, and they communicate/coordinate with each other using industry-standard protocols. Compounds were identified by comparison to library entries of purified standards or recurrent unknown entities. Metabolon maintains a library based on authenticated standards that contains the retention time/index (RI), mass to charge ratio (m/z), and fragmentation data on all molecules present in the library. Furthermore, biochemical identifications are based on three criteria: retention index within a narrow RI window of the proposed identification, accurate mass match to the library +/- 10 ppm, and the MS/MS forward and reverse scores between the experimental data and authentic standards. The MS/MS scores are based on a comparison of the ions present in the experimental spectrum to the ions present in the library spectrum. While there may be similarities between molecules based on one of these factors, the use of all three data points is utilized to distinguish and differentiate biochemicals. More than 5,400 commercially available purified or in-house synthesized standard compounds have been acquired and analyzed on all platforms for determination of their analytical characteristics. An additional 7000 mass spectral entries have been created for structurally unnamed biochemicals, which have been identified by virtue of their recurrent nature (both chromatographic and mass spectral).

#### Compound quality control

A variety of curation procedures were carried out to ensure that a high-quality data set was made available for statistical analysis and data interpretation. The QC and curation processes were designed to ensure accurate and consistent identification of true chemical entities, and to remove or correct those representing system artifacts, mis-assignments, mis-integration and background noise. Metabolon data analysts use proprietary visualization and interpretation software to confirm the consistency of peak identification and integration among the various samples.

#### Metabolite quantification and data normalization

Peaks were quantified using area-under-the-curve. For studies spanning multiple days, a data normalization step was performed to correct variation resulting from instrument inter-day tuning differences. Essentially, each compound was corrected in run-day blocks by registering the medians to equal one (1.00) and normalizing each data point proportionately (termed the “block correction”). For studies that did not require more than one day of analysis, no normalization is necessary, other than for purposes of data visualization. In certain instances, biochemical data may have been normalized to an additional factor (e.g., cell counts, total protein as determined by Bradford assay, osmolality, etc.) to account for differences in metabolite levels due to differences in the amount of material present in each sample.

### Differential metabolite abundance analysis

All analyses were performed using R (v4.5.0). Metabolomics data was batch normalized, imputed, and log transformed by Metabolon. Chemical annotations from Metabolon were enriched by merging with KEGG compound identifiers obtained from Metaboanalyst (v6.0) (46) mapping files using molecule name, Human Metabolome Database (HMDB) (47), and PubChem (48).

Log-transformed metabolite intensity distributions were evaluated for normality using Shapiro-Wilk test (49, 50) from stats (v4.5.1) R packaged and for homogeneity of variance using Levene’s test (51, 52) from car (v3.1-5) R package. Because the data exhibited significant non-normality while maintaining equal variance across groups, statistical analysis for metabolites was performed using rank-based statistical testing adjusting for age group and sex (53, 54). Log-transformed intensities were rank-transformed and fit with a linear model. BH adjusted p-value (55) for each metabolite was reported. BH < 0.05 was considered statistically significant. Fold change was calculated as the mean scaled intensities of the old group divided by the young group.

### Metabolic pathway enrichment analysis

Pathway enrichment analysis was initially performed using the Relational Database of Metabolomics Pathways (RaMP-DB; v3.0.7) (56). To maximize pathway coverage, input metabolites were queried using multiple identifier types, including PubChem (48), HMDB (47), KEGG (25), ChemSpider (57), and Chemical Entities of Biological Interest (ChEBI) (58). We identified pathways associated with the input lists and assessed enrichment using a Fisher’s exact test relative to a blood biospecimen background. Significance was defined as a Holm-adjusted p-value < 0.05.

Due to limited mapping of metabolites to metabolic pathways (mapping rate: ~34% for both accumulated and depleted metabolites), we performed a complementary Metabolite Set Enrichment Analysis (MSEA) to capture broader biological context. This was conducted using the fgsea (v1.32.4) R package (59), utilizing Metabolon subpathways as the *a priori* metabolite sets, which has been used previously in Do et al. (60). Pathways achieving an adjusted p-value < 0.05 were considered significant.

### Integrated KEGG pathway visualizations

KEGG (25) pathway maps were generated to integrate the metabolite differential abundance results with the differential gene expression results using the pathview (v1.48.0) package (61). The Metabolon molecules were mapped to KEGG compounds and for molecules mapping to the same compound, the one with the smallest adjusted p-value value was retained. The differential gene expression gene symbols were mapped to KEGG ortholog identifiers with duplicate entry selection using the same method. Compound and gene differential statistics log_2_FC and log_10_(adjusted p-value) were mapped to KEGG (25) pathway maps. Additional maps not analyzed in this manuscript can be found in the study github repository.

### Supervised bulk RNAseq and metabolomics integration

To identify a robust molecular signature capable of discriminating between senior and young canines, we performed *N*-integration using the DIABLO (27) framework (Data Integration Analysis for Biomarker discovery using Latent variable approaches for Omics studies), implemented in the mixOmics (v6.32.0) R package (62). DIABLO performs supervised classification, maximizing the covariance between multiple omics blocks (bulk transcriptomics and metabolomics) and the categorical outcome (senior vs young) in this study.

First, we applied variance stabilizing transformation (VST) using DESeq2 on transcriptomics data and selected the top 5000 most variable genes (sd > 2) as input. For metabolomics, we selected a matched sample (39 canines) and used Metabolon’s batch of normalized, imputed and log-transformed data as input. Both of these inputs were z-score standardized using DIABLO’s ‘scale = TRUE’ option in both ‘tune.block.splsda’ and ‘block.splsda’.

We performed a systematic design matrix tuning to optimize the trade-off between maximizing the correlation between omics datasets versus maximizing the discrimination of the outcome groups. We evaluated design weights (w) ranging from 0 (prioritizing discrimination) to 1 (prioritizing correlation) in increments of 0.1. For each design weight, we performed a grid-based search using tune.block.splsda to identify the optimal number of features (keepX). The search space included 25, 50, 100, 150, and 200 genes, and 5, 10, 20, 30, and 50 metabolites. This tuning was conducted via 5-fold cross-validation repeated 100 times. Final model performance and feature importance were then validated using 100 iterations of 5-fold cross-validation via the ‘perf’ function. To ensure the robustness of our findings, we conducted a stability analysis using the Jaccard Similarity Index. This metric quantified the overlap of selected features between different design weights. A Jaccard Index close to 1 indicates high stability. While the RNA feature selection was robust across a wide range of weight, the metabolomics signature displayed distinct regions of stability. We observed a consistent ‘stability block’ for metabolomics starting at w = 0.7, where the Jaccard similarity index remained high (> 0.7). The final model parameters (w and keepX) were selected by minimizing the Balanced Error Rate (BER) and maximizing the stability of the selected features. Consequently, a design weight of 0.7 corresponded with lowest BER (RNA = 0.036, metabolomics = 0.233). Final model performance and feature importance were then validated using 100 iterations of 5-fold cross-validation via the ‘perf’ function.

### Weighted gene co-expression network analysis

Gene co-expression networks were constructed from whole blood bulk RNA-seq data using the WGCNA (v1.73) R package (28). Raw count matrices were filtered to retain genes with sufficient expression (counts ≥ 10 in at least 25% of samples; ≥10/39 animals). Filtered counts were variance-stabilized using DESeq2 (vst; blind=TRUE), and the resulting expression matrix was used as input to WGCNA. To focus the network on informative genes and reduce noise, genes were further filtered by robust variability using the median absolute deviation (MAD), retaining the most variable subset (top 50% by MAD; ~6,500 genes).

A signed co-expression network was constructed to preserve the direction of gene–gene relationships. Pairwise gene correlations were computed using biweight midcorrelation (bicor; maxPOutliers = 0.10) and transformed into an adjacency matrix using a soft-thresholding power = 4, chosen using ‘pickSoftThreshold’ based on the scale-free topology fit and mean connectivity. The adjacency matrix was converted to a topological overlap matrix (TOM), and genes were hierarchically clustered using TOM-based dissimilarity (1 − TOM). Modules of co-expressed genes were identified using dynamic tree cutting (deepSplit = 2) with a minimum module size of 30 genes, followed by module merging based on eigengene similarity (merge cut height = 0.25). Module eigengenes (MEs), defined as the first principal component of each module’s gene expression matrix, were calculated and used to summarize module-level expression patterns across samples. Genes not assigned to any module were labeled as “grey” and were excluded from biological interpretation.

The co-expression modules eigengenes were correlated with a focused set of discriminant metabolites identified by DIABLO in the previous step using log-transformed, batch-normalized, and imputed serum metabolite abundances from Metabolon. Module–metabolite associations were assessed using Pearson correlation (ME–trait correlation), and p-values were computed using Student’s asymptotic test as implemented in WGCNA (corPvalueStudent), with multiple testing controlled using FDR. Modules of interest were defined based on the magnitude and consistency of correlations across the DIABLO-selected metabolites.

Functional annotation of modules of interest was performed using pathway ORA. Module gene sets were tested for enrichment for GO-BP terms and KEGG pathways using clusterProfiler with the canine annotation package (org.Cf.eg.db). The background universe was defined as all genes included in the WGCNA analysis (6500 genes). Significance was assessed at FDR < 0.05.

### Cross-species orthology quality classification

Human-canine gene orthology relationships and loss status were obtained from Zoonomia (19). In short, Zoonomia implemented the Tool for Orthologous Gene Annotation (TOGA) (63). TOGA output contains two key files used for analysis: Orthology_classification.tsv, which describes the type of orthologous relationship (e.g., ‘one2one’, ‘one2many’), and Loss_summ_data.tsv, which classifies the intactness of each gene (e.g., ‘I’ for Intact, ‘L’ for Lost).

To create a composite quality metric, we developed a custom grading scheme, Hill’s Grade. This scheme integrates the orthology class with the gene intactness status, assigning a grade from ‘A’ (highest confidence) to ‘F’ (lowest confidence or unclassified) based on the following logic:

- Grade A: Assigned to ‘one2one’ orthologs classified as ‘Intact’ (I).
- Grade B: Assigned to ‘one2one’ orthologs not classified as ‘Intact’, or ‘one2many’/’many2one’ orthologs classified as ‘Intact’.
- Grade C: Assigned to ‘one2many’/’many2one’ orthologs not classified as ‘Intact’, or ‘many2many’ orthologs classified as ‘Intact’.
- Grade D: Assigned to ‘many2many’ orthologs not classified as ‘Intact’.
- Grade F: Assigned to all other cases not meeting the above criteria (e.g., ‘one2zero’ orthology).

For both human-canine and murine-canine gene orthology, only genes graded as ‘A’ were considered further for analysis.

### Integration with open targets

For functional integration, we queried the Open Targets Platform¹¹ for genes associated with the Experimental Factor Ontology term “aging” (EFO_0002507; accessed in November 2025). We stratified targets into three confidence tiers based on GWASCredibleSet metric, which represents an aggregated evidence score derived from multiple independent GWAS studies. We selected a conservative GWASCredibleSet to identify high-confidence candidates (GWASCredibleSet > 0.5). Next, we filtered for those with high-confidence orthologs (Hill’s score = A) and intersected with our canine DEGs (**Supplementary Table 21**).

### Functional integration with IMPC

For IMPC integration, we interpreted concordance between downregulated genes and murine knockout phenotypes as evidence of age-related functional decline. We intersected downregulated high-confidence orthologs with our gene-phenotype pairs (Accessed in June 2024).

Phenotype terms were extracted and parsed from the annotation strings, and duplicate entries per gene were removed to ensure a unique gene-to-phenotype mapping. Enrichment was assessed using a one-sided hypergeometric test (equivalent to Fisher’s exact test) to determine if specific phenotypic terms were overrepresented in the downregulated gene set relative to the background universe. The background universe was defined as the complete set of genes with phenotypic annotations available in the provided IMPC dataset. Final results were filtered to include only terms with a fold enrichment > 0.5 and a proportion of at least 2% regardless of significance.

### Assessment of cell type specificity

To determine the cellular specific expression in the canine GWAS results, we projected the 11 candidate genes onto the single-cell dataset. We utilized the tissue specificity index, Tau, to quantify the lineage-specificity of each gene across the identified cell clusters (64).

For each gene, the Tau score was calculated as:


$$\tau =\;\frac{\sum_{i=1}^{N}(1-\hat{X)}}{N-1}$$

Where N represents the number of cell clusters (27), and $\hat{\left(X\right)}$ is the average normalized expression of the gene in the cluster (i) divided by the maximum expression of that gene across all clusters. This metric ranges from 0 to 1, where a score closer to 1 indicates highly specific expression in a single cell type, and a score closer to 0 indicates broad, ubiquitous expression. Genes were categorized by their primary cell type of origin based on the cluster exhibiting the maximum average expression, however, only values of more than 0.8 were considered cell type specific.

## Data Availability

Raw and processed sequencing data can be found at the NCBI Gene Expression Omnibus (https://www.ncbi.nlm.nih.gov/geo/) under accession numbers (https://www.ncbi.nlm.nih.gov/geo/query/acc.cgi?acc=GSE342969) and (https://www.ncbi.nlm.nih.gov/geo/query/acc.cgi?acc=GSE342551). All analysis and figure-generation code is available upon request to the corresponding author.

## Supplementary material

Supplementary Figure 1 Sub-clustering analysis of the miscellaneous population identified five subsets. Statistical significance was determined using propeller (speckle) with Benjamini–Hochberg FDR adjustment (ns: not significant, *FDR < 0.05, **FDR < 0.01). Young n = 15, Senior n = 15.

Supplementary Figure 2 Comparison of independent PCA for metabolomics and gene expression (top) and integrated DIABLO (bottom) models.

Supplementary Figure 3 Celltype specificity for canine GWAS signals.
