# Supplementary material for: Multiomic profiling of canine immune and metabolic aging in a cohort of young and senior beagles
Source: Front Immunol. 2026 Sep 8;17:1906638. doi: 10.3389/fimmu.2026.1906638 (PMC13593421; doi:10.3389/fimmu.2026.1906638)
Supplement: Supplementary file 1 [file Image1.pdf]

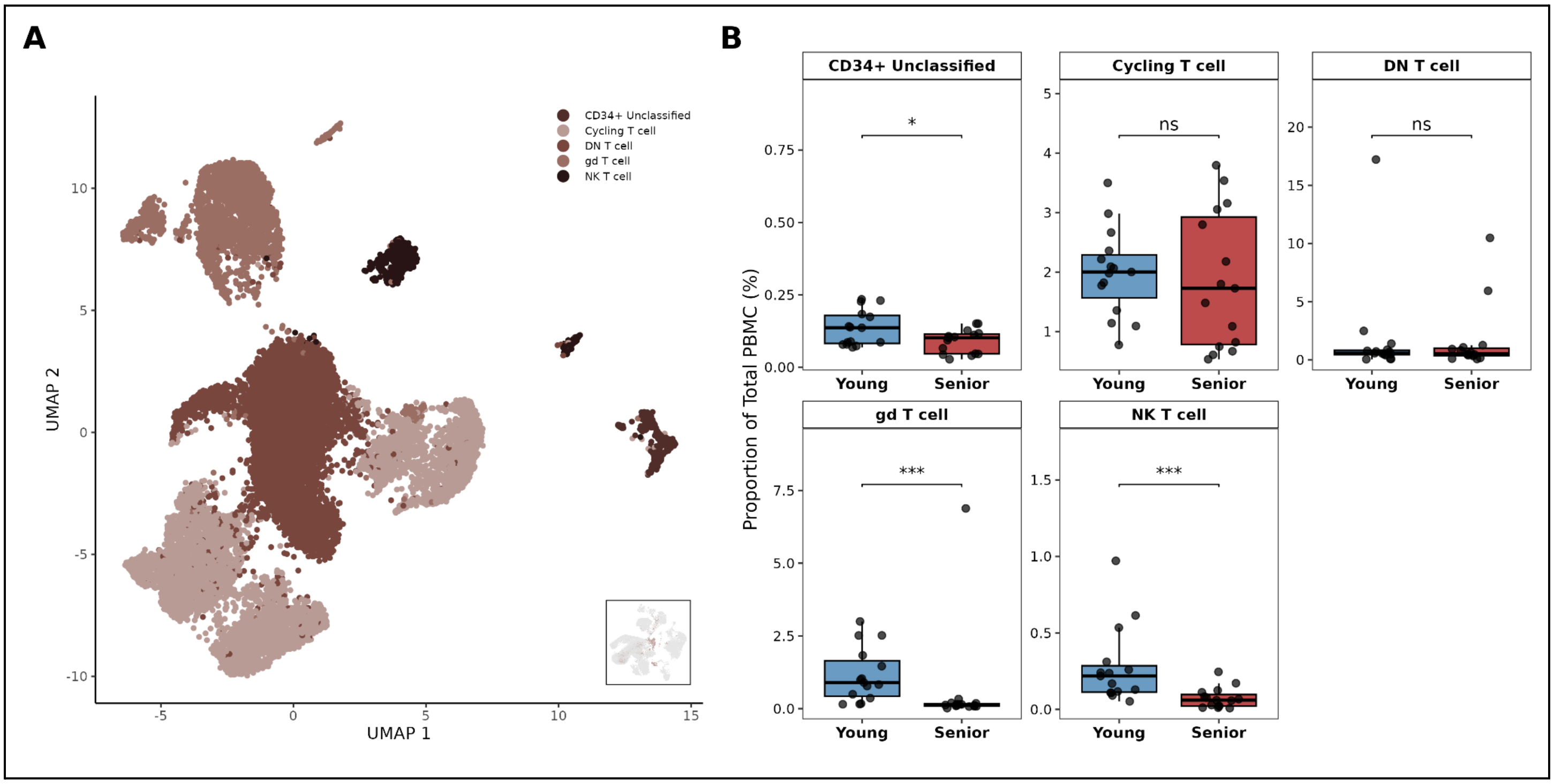

Figure S1. Sub-clustering analysis of the miscellaneous population identified five subsets. Statistical significance was determined using propeller (speckle) with Benjamini–Hochberg FDR adjustment (ns: not significant, *FDR < 0.05, FDR < 0.01). Young n = 15, Senior n = 15.
