# Supplementary material for: Multiomic profiling of canine immune and metabolic aging in a cohort of young and senior beagles
Source: Front Immunol. 2026 Sep 8;17:1906638. doi: 10.3389/fimmu.2026.1906638 (PMC13593421; doi:10.3389/fimmu.2026.1906638)
Supplement: Supplementary file 3 [file Image3.pdf]

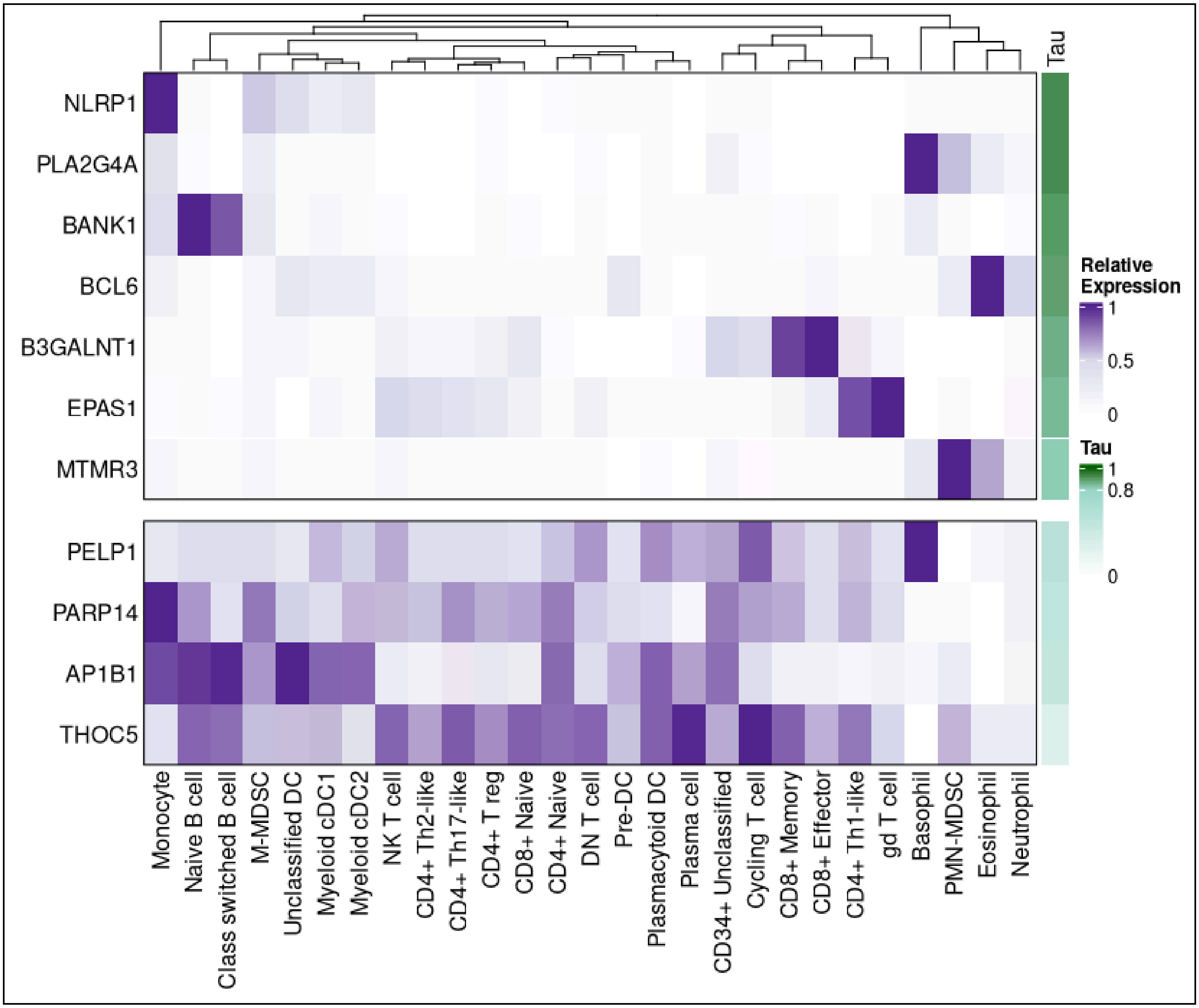

Figure S3. Celltype specificity for Canine GWAS signals
